# A circular RNA *Edis*-Relish-*castor* axis regulates neuronal development in *Drosophila*

**DOI:** 10.1371/journal.pgen.1010433

**Published:** 2022-10-27

**Authors:** Wei Liu, Weihong Liang, Xiao-Peng Xiong, Jian-Liang Li, Rui Zhou

**Affiliations:** 1 Department of Medicine, Johns Hopkins University School of Medicine, Baltimore, Maryland, United States of America; 2 Department of Biological Chemistry, Johns Hopkins University School of Medicine, Baltimore, Maryland, United States of America; 3 Department of Oncology, Johns Hopkins University School of Medicine, Baltimore, Maryland, United States of America; 4 Cancer and Blood Disorders Institute, Johns Hopkins All Children’s Hospital, St. Petersburg, Florida, United States of America; 5 Institute for Fundamental Biomedical Research, Johns Hopkins All Children’s Hospital, St. Petersburg, Florida, United States of America; 6 Tumor Initiation and Maintenance Program, NCI-Designated Cancer Center, Sanford Burnham Prebys Medical Discovery Institute, La Jolla, California, United States of America; 7 Development, Aging and Regeneration Program, Sanford Burnham Prebys Medical Discovery Institute, La Jolla, California, United States of America; 8 National Institute of Environmental Health Sciences, Durham, North Carolina, United States of America; University of Michigan, UNITED STATES

## Abstract

Circular RNAs (circRNAs) are a new group of noncoding/regulatory RNAs that are particularly abundant in the nervous system, however, their physiological functions are underexplored. Here we report that the brain-enriched circular RNA *Edis* (*Ect4*-*d*erived *i*mmune *s*uppressor) plays an essential role in neuronal development in *Drosophila*. We show that depletion of *Edis in vivo* causes defects in axonal projection patterns of mushroom body (MB) neurons in the brain, as well as impaired locomotor activity and shortened lifespan of adult flies. In addition, we find that the *castor* gene, which encodes a transcription factor involved in neurodevelopment, is upregulated in *Edis* knockdown neurons. Notably, *castor* overexpression phenocopies *Edis* knockdown, and reducing *castor* levels suppresses the neurodevelopmental phenotypes in *Edis*-depleted neurons. Furthermore, chromatin immunoprecipitation analysis reveals that the transcription factor Relish, which plays a key role in regulating innate immunity signaling, occupies a pair of sites at the *castor* promoter, and that both sites are required for optimal *castor* gene activation by either immune challenge or *Edis* depletion. Lastly, *Relish* mutation and/or depletion can rescue both the *castor* gene hyperactivation phenotype and neuronal defects in *Edis* knockdown animals. We conclude that the circular RNA *Edis* acts through Relish and *castor* to regulate neuronal development.

## Author summary

Circular RNAs (circRNAs) are a new group of noncoding/regulatory RNAs that are particularly abundant in the nervous system, although their physiological functions are underexplored. Here we report that the brain-enriched circular RNA *Edis* (*Ect4*-*d*erived *i*mmune *s*uppressor) plays an essential role in neuronal development in the fruitfly *Drosophila melanogaster*, as its depletion causes defects in the development of neurons in a brain structure called mushroom body (MB), as well as impaired locomotor activity and shortened lifespan of adult flies. In addition, we show that the *castor* gene, which encodes a protein involved in neurodevelopment, is upregulated in *Edis* knockdown neurons. Notably, flies with increased levels of *castor* or reduced levels of *Edis* display similar phenotypes, and reducing *castor* levels rescues the developmental defects in *Edis*-depleted neurons. Furthermore, we find that the immune regulator Relish occupies a pair of sites at the *castor* gene locus, and that both sites are required for optimal *castor* gene activation by either immune challenge or *Edis* depletion. Lastly, *Relish* mutation and/or depletion can rescue both the *castor* gene hyperactivation phenotype and neuronal defects in *Edis* knockdown animals. We conclude that the circular RNA *Edis* acts through Relish and *castor* to regulate neuronal development.

## Introduction

Circular RNAs (circRNAs) are the latest addition to the noncoding and regulatory RNA collection. They are characterized as covalently closed RNA loops generated by “head-to-tail” back-splicing events [1,2]. With the development of high-throughput sequencing technologies and bioinformatic approaches, thousands of circRNAs have been identified in a wide variety of eukaryotic organisms including human, mouse, worm, and fruit fly [3–6]. Subsequent functional studies have implicated select circRNAs in various physiological and pathological processes, including testes development [7,8], cell cycle progression [9], cancer-associated cell proliferation [10,11], and neuropsychiatric disorders [12,13]. Circular RNAs can function as regulators of microRNA biogenesis/function, operate as scaffold for the assembly of protein/RNA complexes, or modulate host gene expression. Recently, select circRNAs have been shown to encode functional proteins [14,15]. Since circRNAs generally lack a 5’ cap and poly(A) tail, translation of circRNAs is mediated by cap-independent mechanisms, including internal ribosome entry site (IRES) or N6-methyladenosine (m6A) mediated ribosome recruitment [1,16,17,18].

While circRNAs are present across most cell/tissue types [19–21], they are particularly abundant in the nervous system [3,22–26], suggesting a key role in neurodevelopment. Indeed, a handful of neuronal circRNAs have been functionally characterized. For example, *CDR1as* binds to *microRNA-7* (*miR-7*) and regulates *miR-7* biogenesis, thereby impacting brain development [7,8,12,27]. In addition, *circZNF827* functions as a scaffold for a transcription repressive complex containing ZNF827, hnRNP K, and hnRNP L to regulate neuronal differentiation [28]. Furthermore, the psychiatric disease-associated circRNA *circHomer1a* interacts with the HuD protein and further influences *HuD* gene expression in the frontal cortex [13]. Despite these well-characterized circRNAs, our knowledge of how circRNAs are involved in neuron development/function is still limited. Thus, it is important to identify and functionally characterize additional neuronal circRNAs in healthy and disease settings, and to elucidate the underlying mechanisms.

Innate immunity, the first line of defense, protects hosts against invading microbes and adverse effect of stress signals generated by injured cells. While rapid and robust activation of the innate immune response is crucial for host fitness, aberrant or prolonged activation can cause detrimental consequences. For example, aging and neurodegenerative conditions are often associated with aberrant activation of immunity signaling [29]. In addition, long-term pharmacological suppression of the inflammatory response can lead to a reduction in risk of developing neurodegenerative diseases [30,31]. Thus, the magnitude and duration of innate immunity activation need to be tightly controlled in order to maintain a delicate balance between host defense and nervous system integrity/function.

*Drosophila melanogaster* is a powerful model organism to advance our understanding of the molecular mechanism underlying innate immunity activation. Upon encountering diaminopimelic acid (DAP)-type peptidoglycan (PGN), a cell wall component derived from Gram-negative and certain Gram-positive bacteria, a dedicated IMD (immune deficiency) signaling pathway is activated [32]. The IMD pathway involves membrane-bound receptor PGRP-LC; adaptor molecules IMD and dFADD; ubiquitination enzymes Bendless, dUEV1a and dIAP-2; protein kinase complexes dTAK1/dTAB2 and Ird5/Kenny; and the caspase Dredd, culminating in the proteolytic processing and activation of the NF-κB family transcription factor Relish, nuclear translocation of the N-terminal fragment of Relish, and activation of genes encoding a battery of antibacterial peptides [33–36]. Similar to the observations made in humans, aberrant activation of innate immunity in *Drosophila* can result in phenotypes indicative of neurodegeneration. For example, depletion of ATM (AT mutated) in glial cells causes elevated expression of innate immune response genes in glial cells as well as neuronal and glial cell death, and a reduction in mobility and longevity [37]. In addition, it has been reported that flies with mutations in *dnr1* (defense repressor 1) exhibit shortened lifespan and progressive, age-dependent neuropathology associated with aberrant activation of the IMD pathway and elevated expression of antimicrobial peptide (AMP) genes [38]. Furthermore, ectopic expression of individual AMP genes in the *Drosophila* brain results in brain damage [38]. These findings highlight the connection between dysregulated innate immunity signaling and neurodegeneration in flies.

In a recent study, we describe the identification and functional characterization a circRNA *circEct4*, also known as *Edis* (*Ect4-derived immune suppressor*) [39]. Knockdown of *Edis*, but not its linear sibling *Ect4*, specifically in neurons causes hyperactivation of innate immunity and myriad defects in neuronal development. We show that *Edis* can be translated into a functional protein Edis-p, which binds to, and compromises, the proteolytic processing/activation of the immune transcription factor Relish. In addition, inactivation of *Relish* in *Edis*-depleted neurons rescues the innate immunity hyperactivation phenotype, suggesting that Relish acts downstream of *Edis* to regulate immunity. However, the detailed mechanism underlying the function of *Edis* in regulating the neurodevelopment is still elusive, and the target/effector gene(s) downstream *Edis*/Relish remain to be identified and functionally characterized.

Here, we report that *Edis* is critically required for mushroom body (MB) development in *Drosophila*. We show that the *Edis* transcript is enriched in neurons, consistent with its role in neuronal development. Notably, loss of *Edis* leads to axonal misguidance in MB neurons and elevated expression of *castor*, which encodes a transcription factor critical for neuronal development. Importantly, overexpression of *castor* phenocopies *Edis* depletion, and reducing *castor* levels in *Edis*-depleted neurons rescues defects in MB morphology, locomotor activity and lifespan. We provide evidence that the immune transcription factor Relish binds to the *castor* promoter and regulates *castor* transcription. Thus, our study reveals a crucial function of the circRNA *Edis* in regulating MB neuronal development in *Drosophila*, establishes *castor* as an effector/target gene downstream of *Edis*, and generates an animal model that can facilitate unraveling of intricate interplay between innate immunity signaling and neuronal development.

## Results

### *Edis* is required for mushroom body neuronal development

**Fig 1:**
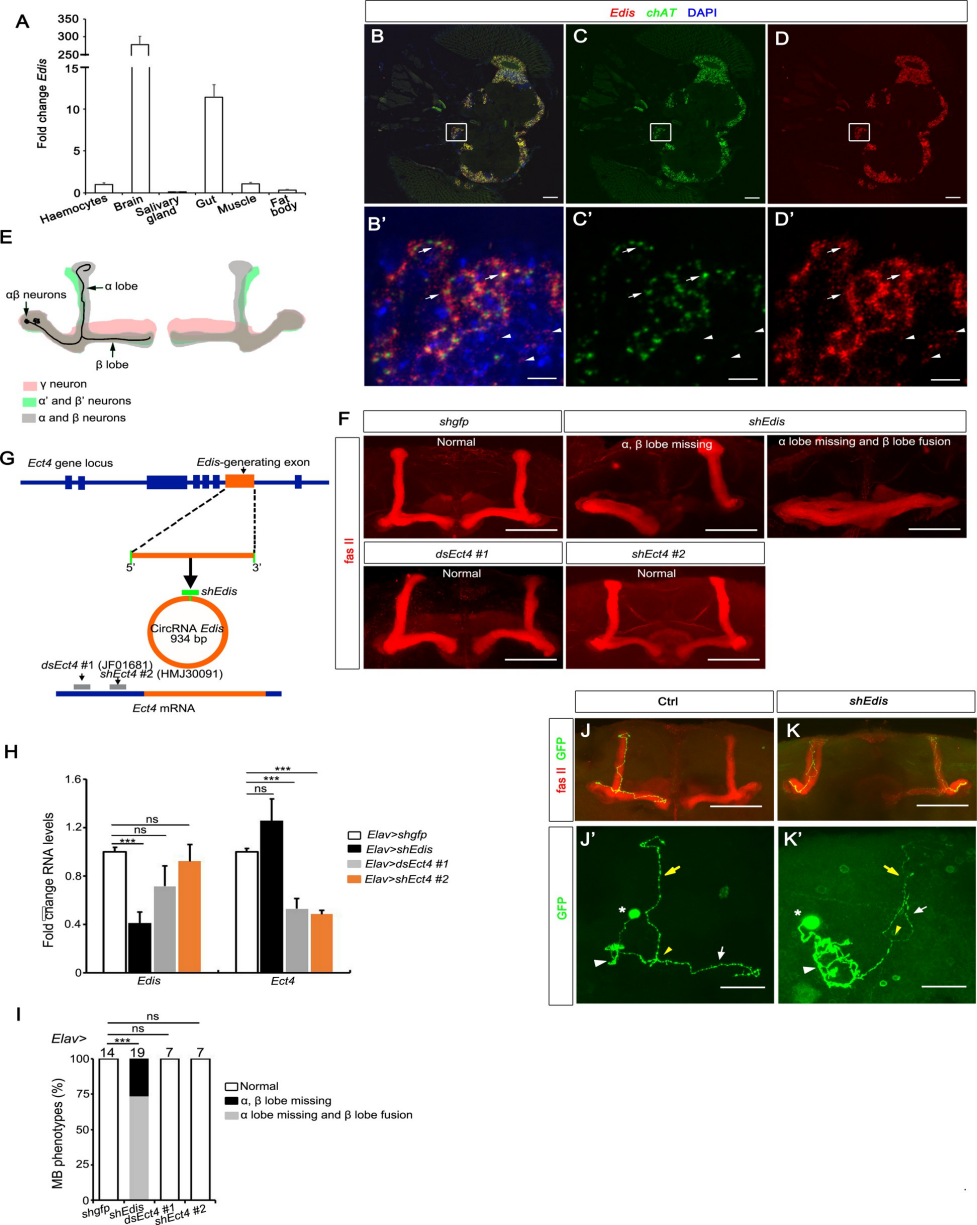
*Edis* is required for mushroom body development. (**A**) Levels of *Edis* in various tissues from the *Drosophila* 3^rd^ *instar* larvae were measured by real-time PCR (n = 3). *Edis* is highly expressed in the brain. Data are presented as mean + SEM. *p<0.05; **p<0.01. (**B-D’**) Fluorescence in situ hybridization (FISH) analysis of various RNAs in adult fly brains. *Edis* (red) is widely distributed in neurons. The mRNA encoding Choline acetyltransferase (*ChAT*, green) labels cholinergic neurons. *Edis* is expressed in both *chAT*-positive (top 3 arrows) and *chAT*-negative (bottom 3 arrowheads) neurons. Nuclei were marked by DAPI (blue). **C** and **D** are split channels of **B**. **B’-D’** show high magnification images of boxed regions in **B-D**. The scale bars indicate 50 μm in **B**-**D** and 5 μm in **B’-D’**. (**E**) A diagram showing the three types of MB neurons that display characteristic projection patterns to form distinct axonal bundles. The MB αβ neurons are in grey, α’β’ neurons in green, and γ neurons in pink. Individual MB αβ neurons extend their axons along the peduncle and the axons bifurcate, forming a dorsal (α lobe) and a medial (β lobe) branch. (**F**) Fasciclin II (Fas II) antibody staining labels MB αβ neurons. Images in various panels show normal and defective MB morphology of the indicated genotypes. Scale bars indicate 50 μm. (**G**) A diagram showing the *Ect4* mRNA and *Edis* cricRNA derived from the *Ect4* locus as well as the mapping of various shRNA/dsRNA reagents. (**H**) Levels of *Edis* and *Ect4* RNAs in fly brains of various genotypes were measured by real time RT-PCR (n = 3–12). (**I**) Quantification of MB phenotypes in flies of indicated genotypes. *Edis* depletion led to MB morphology defects, whereas loss of *Ect4* had no impact. Chi-squared test was employed in statistical analysis. Sample numbers are shown on top. (**J-K’**) Single MB neurons were labeled using the MACRM technique in the indicated genotypes. Fas II (red) labels the MB αβ axon, and GFP (green) labels the MB αβ neuron. **J’** and **K’** show high magnification single GFP channel images in **J** and **K**. MB αβ neuron cell body (indicated by star), dendrites (white arrowhead), branching point (yellow arrowhead), α lobe (yellow arrow), β lobe (white arrow) are shown. The missing β lobe phenotype in *Edis* knockdown fly brains (**K**) is a result of axon misguidance to the α lobe direction (**K’**). The scale bars represent 50 μm and 20 μm, respectively, in **J-K** and **J’-K’**.

To gain a full insight into the function of the circular RNA *Edis in vivo*, we first analyzed the distribution pattern of *Edis* in *Drosophila melanogaster*. Because the tissues/organs of *Drosophila* larvae can be easily distinguished and separated, different tissues/organs of third *instar* larvae were collected and levels of *Edis* were examined. Among the tissues/organs examined, including hemocytes, salivary gland, gut, body wall muscle, and fat body, *Edis* was most prominently enriched in the brain (**Fig 1A**). This is consistent with the notion that circRNAs are enriched in the nervous system [22,23,39]. To further visualize the localization pattern of *Edis* within the brain, RNA fluorescence in situ hybridization assay was performed. Specifically, an RNA probe complementary to sequences at the unique “back-spliced” exon junction was designed to label *Edis*, which can specifically recognizes *Edis* (**S1A–S1F’ Fig**). A control probe was designed against *chAT* (*Choline acetyltransferase*) transcript, an enzyme required for the biosynthesis of the neurotransmitter acetylcholine, to label cholinergic neurons [40]. In both *chAT*-positive and -negative neurons, *Edis* was predominantly localized in the cytosol (**Fig 1B** and **1D**). Interestingly, *chAT* and *Edis* display similar expression patterns (**Fig 1B and 1D**), suggesting a potential role of *Edis* in cholinergic neurons in the *Drosophila* CNS.

Mushroom body (MB) neurons are a group of cholinergic neurons in the *Drosophila* CNS [41] that function in olfactory learning and memory [42]. One MB contains ~2000 Kenyon cells. Each cell body sends out one primary neurite which gives rise to dendrites and then extends axons branching out ventrally and anteriorly through the peduncles. At the end of peduncles, three types of sequentially formed MB axons are segregated into three distinct sets of lobes, namely the γ lobe, α’ and β’ lobes, and α and β lobes (**Fig 1E**) [43]. The α and β lobes can be recognized for their high levels of FasII expression as in contrast to the much weaker FasII expression in the γ lobe, thus the characteristic morphology of MB can be easily recognized (**Fig 1F**). Given the high level of *Edis* expression in the cholinergic neurons, we focused on investigating the role of *Edis* in MB neuron development in detail. First, we depleted *Edis* by RNA interference (RNAi) in neurons using a pan-neuronal Gal4 driver, *Elav-Gal4*, for targeted expression of short hairpin RNA against *Edis* (*shEdis)* (**Fig 1G**). This manipulation resulted in a ~60% reduction of *Edis* levels in *shEdis*-expressing neurons, whereas levels of linear sibling *Ect4* transcripts were comparable with those in control *Elav>shgfp* animals (**Fig 1H**). Strikingly, such *Edis* knockdown resulted in severe MB phenotypes, including partial or complete absence of α and β lobes, or absence of α lobe accompanied with β lobe fusion (**Fig 1F and 1I**). In contrast, all the *Ect4* knockdown (*Elav>dsEct4* and *Elav>shEct4*) brains showed normal MB morphology (**Fig 1F–1I**). *Ect4* has been previously implicated in neurodevelopment and neuronal cell death upon injury [44], It is possible that the absence of apparent MB morphology defects in *Ect4* knockdown animals could be due to a moderate degree of *Ect4* knockdown efficiency in both *Elav>dsEct4* and *Elav>shEct4* flies (**Fig 1H**).

Recently, we show that overexpression of *Edis* suppressed the IMD innate immunity signaling pathway both in cultured cells and *in vivo* [39]. In addition, we crossed *Elav-Gal4* with *UAS-laccase2-Edis* flies to drive *Edis* expression in neurons. We find that restoring *Edis* expression can rescue the neurodevelopmental phenotypes elicited by *Edis* knockdown [39], thereby demonstrating the functional relevance of overexpressed *Edis*. We therefore employed a similar experimental setting to examine whether overexpressed *Edis* impacts neuronal development. Our analysis reveals that *Edis* overexpression alone did not impact MB morphology (**S1G–S1I Fig**). Taken together, these data demonstrate that *Edis* is enriched in neurons and required for MB neuron development.

The MB morphology phenotype in *Edis*-depleted brains could be due to either axon malformation or defective axon projection to other brain areas. To differentiate these possibilities, we employed the MARCM technique to label individual α/β neurons and their axonal projections [43]. In control samples, mCD8GFP labeled a single MB α/β neuron which branched its axon dorsally along the α lobe and medially along the β lobe (**Fig 1J and 1J’**). In *Edis*-depleted brains, the α/β neuron was still projecting along the peduncle and forming two branches. However, instead of the β axon extending along the medial direction, both branches projected to the dorsal direction, forming a thicker α lobe (**Fig 1K and 1K’**). These results indicate that *Edis* is required for proper axon projection patterns of the MB neurons.

### Loss of *Edis* in the MB neuron precursors results in defective MB morphology

**Fig 2:**
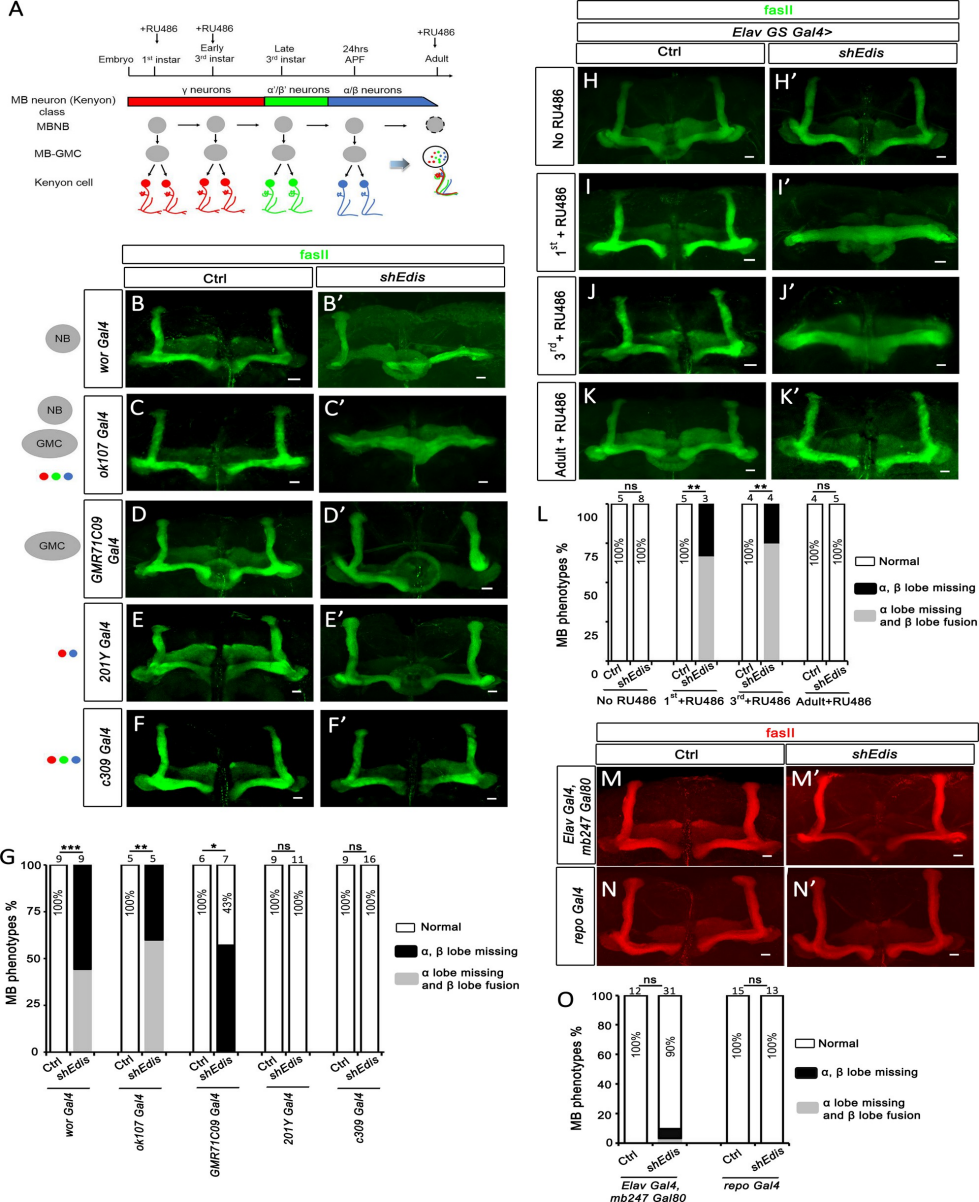
Loss of *Edis* in the MB neuron precursors results in MB morphology defects. (**A**) A diagram showing the MB neuron developmental process during the *Drosophila* lifecycle (modified from [43,48]). MB neuroblasts (MBNB) keep dividing to produce the MB ganglion mother cells (GMC) and further produce mature MB neurons (Kenyon cells) until the adult stage, in which MBNB undergoes apoptosis [51]. (**B**-**F’**) The MB morphology phenotypes in flies of indicated genotypes. MB morphology defects were observed when *UAS-shEdis* was driven by *wor*-*Gal4* (**B**, **B’**), *ok107*-*Gal4* (**C**, **C’**), and *GMR71C09*-*Gal4* (**D**, **D’**). These drivers can induce transgene expression in the MB neuron precursors (MBNB and/or MB-GMC). In contrast, MB morphology is normal when *UAS-shEdis* was driven by *201Y-Gal4* (**E**, **E’**) or *c309-Gal4* (**F**, **F’**), which induces transgene expression only in mature MB neurons. The scale bar indicates 20 μm. (**G**) Quantification of MB morphology phenotypes with indicated genotypes in **B**-**F’**. Chi-squared test was employed in statistical analysis. Sample numbers and percentages of samples showing normal MB morphology in each genotype are shown. (**H**-**K’**) RU486 was added into fly food at different time points as indicated in **A** to activate *Elav-Gene switch (GS)-Gal4*, which in turn drives transgene (shRNA) expression. Fly brains exhibited MB (green) morphology defects when RU486 was added at the 1^st^ (**I**, **I’**) or 3^rd^ (**J**, **J’**) *instar* larval stages. In the absence of RU486 (**H**, **H’**) or when RU486 was added at the adult stage (**K**, **K’**), only normal MB morphology was observed. The scale bar indicates 20 μm. (**L**) Quantification of MB morphology phenotypes with indicated genotypes in **H**-**K’**. Chi-squared test was employed in statistical analysis. Sample numbers are shown on top. (**M** and **M’**) *Elav-Gal4* drives *UAS-shgfp* or *UAS-shEdis* expression in all neurons except MB neurons in which *mb247-Gal80* inhibits *Gal4*. The scale bar indicates 20 μm. (**N** and **N’**) *Edis* depletion in glia using *repo-Gal4* did not lead to MB morphology defects. The scale bar indicates 20 μm. (**O**) Quantification of MB morphology phenotypes with indicated genotypes in **M**-**N’**. Chi-squared test was employed in statistical analysis. Sample numbers and percentages of samples showing normal MB morphology in each genotype are shown.

To further dissect the role of *Edis* in MB formation, we took advantage of a collection of Gal4 drivers to knock down *Edis* at various stages of MB neuron development (**Fig 2A–2F’**). First, *worniu-Gal4 (wor-Gal4)* was employed to drive *Edis* depletion in all neuroblasts (MB-NB) of the *Drosophila* brain [45]. Approximately 44% of the progeny showed MB α lobe missing with β lobe fusion, while the remaining 56% showed α and β lobes missing phenotypes (**Fig 2B, 2B’** and **2G**). To gain more details, we next tested *ok107-Gal4*, which is a pan-MB neuronal driver that remains active from the neuroblast (NB) stage to until the differentiated mature neuron stage [46, 47]. Upon *ok107-Gal4*-driven *Edis* depletion, progeny flies showed profound missing MB α/β lobe and β lobe fusion phenotypes (**Fig 2C, 2C’** and **2G**). Next, *Edis* was depleted by *GMR71C09-Gal4*, which is active predominantly in the MB-ganglion mother cells (MB-GMCs) and early-born neurons [48]. In this cross, ~57% of the progeny showed missing α and β lobes phenotype, whereas the remaining 43% displayed normal MB morphology (**Fig 2D, 2D’** and **2G**). Thus, it appears that even though depletion of *Edis* in the MB-GMCs can lead to MB αβ axonal misprojection, the phenotype was moderate compared with that of flies with *Edis* knockdown at earlier (MB-NB) developmental stages. In contrast, when the knockdown of *Edis* was driven by either *201Y-Gal4* or *c309-Gal4*, which are active only in differentiated mature MB neurons [49], all progeny flies displayed normal MB α/β axon distribution pattern (**Fig 2E–2G**). Overall, these data indicate that *Edis* regulates MB formation at developmental stage(s) (NBs and GMCs) prior to the completion of MB neuron differentiation. To confirm these findings, we next employed *Gene-switch (GS)-Gal4*, a chemical (RU486) dependent inducible UAS/Gal4 system, to precisely control the onset of *Edis* depletion in the *Drosophila* CNS at different developmental stages [50]. Specifically, *Elav GS-Gal4* was crossed to *UAS-shEdis* animals. Subsequently, RU486 was added to fly food at various time points, and MB morphology of adult progeny was examined (**Fig 2A,** and **2H–2K’**). When *Elav GS-Gal4* was activated at the larval stage (*1*^*st*^ *instar* and *3*^*rd*^ *instar*), the MB morphology phenotypes were observed (**Fig 2I–2J’** and **2L**). In contrast, we did not detect any MB defects with the addition of RU486 at the adult stage (**Fig 2K, 2K’** and **2L**), as MB-NBs are eliminated via apoptosis before eclosion [51]. Based on these orthogonal analyses, we conclude that *Edis* is required in developing MB neurons, but not in mature, postmitotic MB neurons for αβ axonal guidance.

We next examined whether *Edis* from non-MB neurons is required for MB formation. The *mb247-Gal80* transgene was employed to suppress *Gal4* activity exclusively in MB neurons [52], whereas *Edis* is depleted in all other types of neurons by *Elav-Gal4*. This analysis revealed that depletion of *Edis* in all non-MB neurons resulted in much milder phenotypes (>90% normal MB morphology) than pan-neuronal *Edis* depletion (no normal MBs) (compare **Figs 1I, 2M, 2M’** and **2O**). We conclude that the MB morphology phenotypes were mediated predominantly by *Edis* depletion in MB neurons. As for the mild MB morphology phenotype (9.7%) observed in *mb247-Gal80*; *Elav>shEdis* animals, it could be due to 1) incomplete inhibition of Gal4 by Gal80, and/or 2) minor contribution of *Edis* from non-MB neurons in the *Drosophila* CNS that impacts MB formation.

Glial cells are an integral component of the nervous system and interact extensively with neurons. Our recent study reveals that *Edis* is expressed in glia [39]. We therefore depleted *Edis* in glial cells using *Repo-Gal4* [53]. All *Repo>shEdis* animals showed normal MB morphology (**Fig 2N–2O**). These results suggest that *Edis* from the MB neuron precursors, but not that from glia, is crucial for proper MB development.

### The neurodevelopmental phenotypes elicited by *Edis* depletion depend on Relish

**Fig 3:**
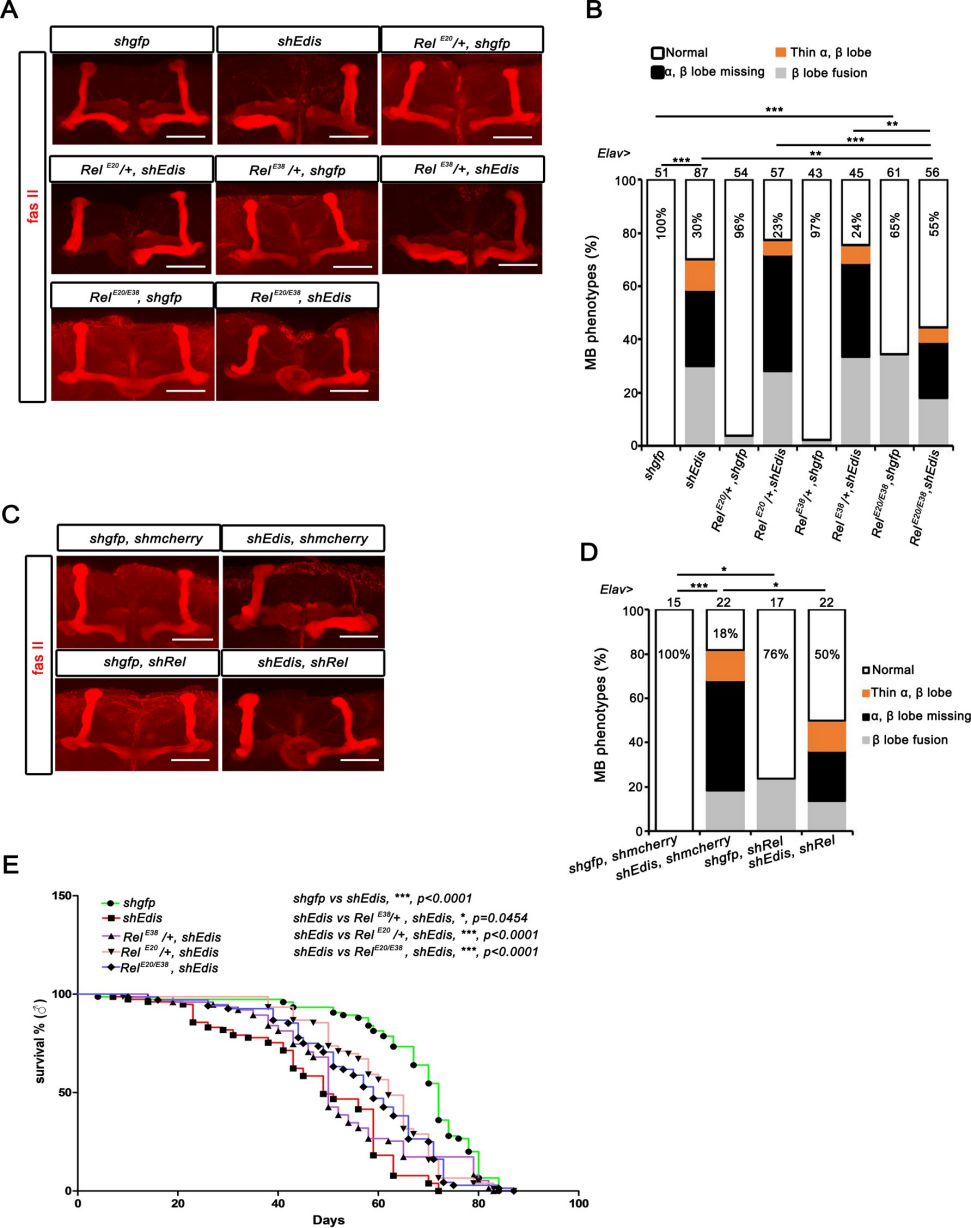
Neuronal phenotypes elicited by *Edis* depletion depend on Relish. (**A**) Confocal images of dorsal anterior regions of adult brains with various MB morphology phenotypes. Depletion of *Edis* in neurons resulted in a spectrum of severe morphological defects in the MBs as noted above each panel. The scale bar indicates 50 μm. (**B**) MB morphology phenotypes were quantified in flies with the neuron-specific expression of control *shgfp* or *shEdis* driven by the *Elav-Gal4* driver in wildtype, Relish heterozygous (*Rel*^*E20*^/+ or *Rel*^*E38*^*/+*) or homozygous (*Rel*^*E20/E38*^) mutant background. Chi-squared test was employed in statistical analysis. Sample numbers and percentages of samples showing normal MB morphology in each genotype are shown. (**C-D**) MB morphology phenotypes of in flies with neuron-specific expression of various combinations of shRNA transgenes (control *shgfp*, *shEdis* and *shRel*) driven by the *Elav-Gal4* driver are shown in **C** and quantified in **D**. Chi-squared test was employed in statistical analysis. Sample numbers and percentages of samples showing normal MB morphology in each genotype are shown. (**E**) Lifespan of flies of select genotypes in **B** is shown.

We found that *Edis* depletion in the *Drosophila* CNS leads to activation of the IMD innate immunity signaling pathway, with dramatically elevated expression of several AMP genes that are normally regulated by Relish, a key immune transcription factor (**S2 Fig**). To investigate the relationship between the neuronal defects and the immunity hyperactivation phenotypes, we examined the impact of *Relish* mutation on the neuronal phenotypes of *Edis* knockdown animals. Consistent with recent reports that implicate *Relish* in neurodevelopment [54,55], we found that about a third of Relish null mutants display β lobe fusion phenotypes (**Fig 3A and 3B**). Despite of this, in *Relish* null mutant background, the MB morphology phenotype in *Elav>shEdis* flies was (at least partially) rescued (**Fig 3A and 3B**, compare *Rel*^*E20/E38*^ with *Rel*^*E20*^*/+*, *Rel*^*E38*^*/+* or *+/+* genetic background). Importantly, a similar observation was made upon depletion of *Relish* in neurons (**Fig 3C and 3D**), demonstrating a cell autonomous interaction between *Relish* and *Edis* in regulating neurodevelopment. Lastly, the lifespan phenotype was also affected by *Relish* mutation (**Fig 3E**). We note that the lifespan differences are complex phenotypes and most likely do not result from Kenyon cell alterations, and that lifespan of *Edis* knockdown flies was also affected by genetic background (**Fig 3E**, compare *Rel+/+* with *Rel*^*E20*^*/+* and *Rel*^*E38*^*/+*). Nonetheless, taken together, data from our analysis on MB morphology in various combinations of *Edis* and *Relish* mutant/knockdown backgrounds strongly suggest that the neurodevelopmental phenotypes elicited by *Edis* depletion depend (at least partially) on Relish.

### *Castor* is upregulated upon neuronal *Edis* depletion

**Fig 4:**
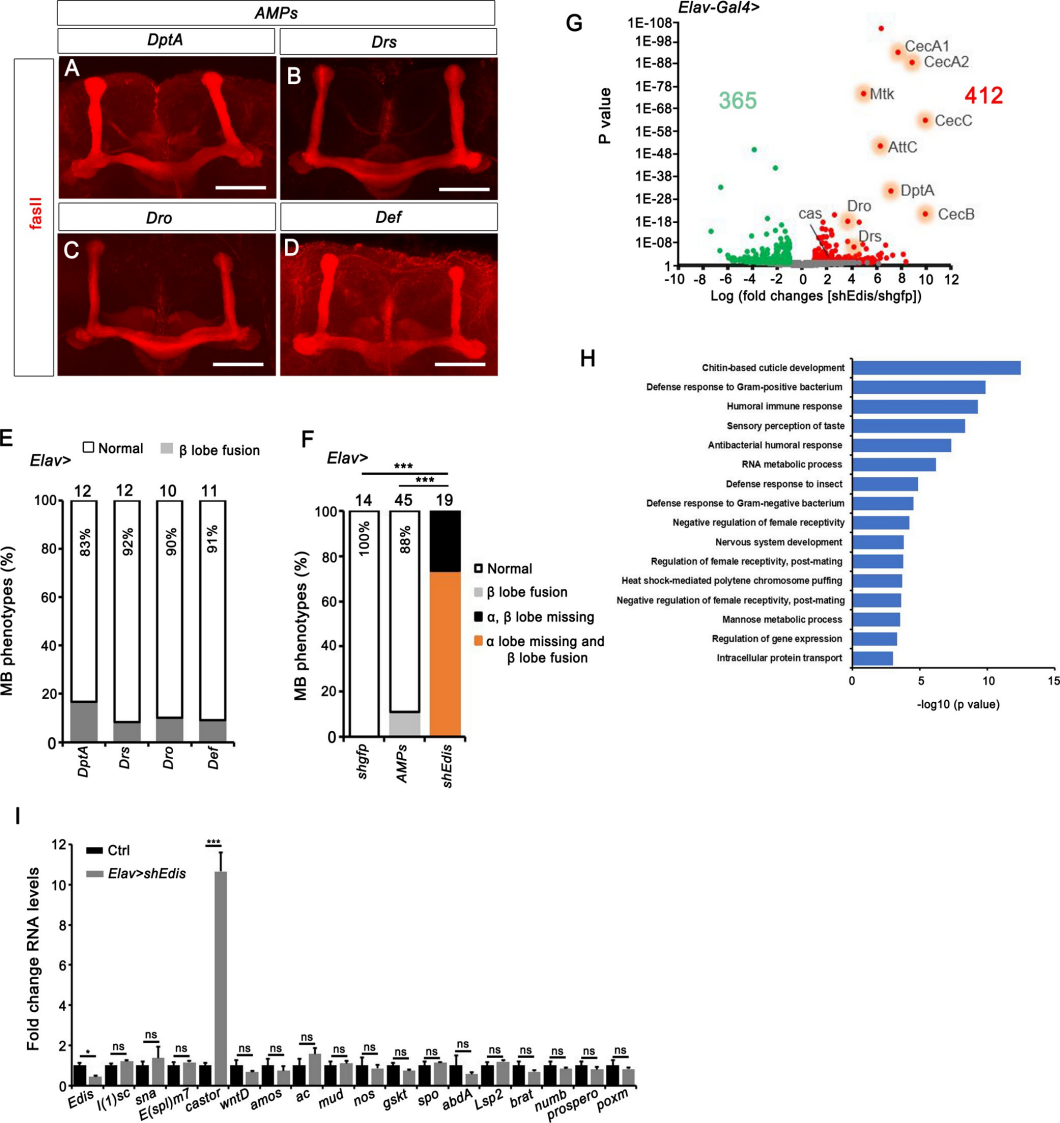
*castor* is upregulated upon neuronal *Edis* depletion. (**A**-**D**) Images showing typical MB morphologies in flies with individual AMP overexpression. The scale bar indicates 50 μm. (**E**) MB morphology phenotypes were quantified in flies with overexpression of AMP genes *DptA*, *Drs*, *Dro*, and *Def* in neurons. Sample numbers and percentages of samples showing normal MB morphology in each genotype are shown. (**F**) MB morphology phenotypes were quantified in flies with the indicated genotypes (neuron-specific expression of control *shgfp*, *shEdis* or AMP genes driven by the *Elav-Gal4* driver). Note that the *shgfp* and *shEdis* data are identical to those shown in **Fig 1I**. We consolidated the individual data points from **F** as *Elav>AMPs*. Chi-squared test was employed in statistical analysis. Sample numbers and percentages of samples showing normal MB morphology in each genotype are shown. (**G**) A volcano plot of gene expression profile of *Elav>shEdis* brain samples compared to wild type. Significantly up- (red, *p* ≤ 0.05 and logFC > 1) and down-regulated genes (green, *p* ≤ 0.05 and logFC ≤ -1) are shown. (**H**) The significantly changed genes were subjected to gene ontology (GO) enrichment analysis. Shown are major biological processes they are involved in. (**I**) RNA levels of select genes involved in neurodevelopment were measured by real-time PCR in *Elav>shEdis* brains and compared with control. *casto*r is significantly upregulated in *Edis* depleted brain tissue (n = 3).

It has been reported that microbial infection or ectopic expression of AMP genes can lead to neurodegeneration [38]. To examine whether forced expression of AMP genes in the brain can lead to defects in MB morphology, we expressed individual AMP genes, including *Diptericin A (DptA)*, *Drosocin* (*Dro*), *Defensin* (*Def*), and *Drosomycin* (*Drs*) in neurons using *Elav-Gal4*. Interestingly, while a fraction of animals with ectopic expression of any of the four AMP genes displayed defective MB morphology, the phenotype is far milder than that seen in *Elav>shEdis* animals, as only 10–18% *Elav>AMP* animals showed MB morphology defects (**Fig 4A–4F**). In addition, only β lobe fusion, but no missing αβ lobe phenotypes were observed in *Elav>AMP* animals (**Fig 4A–4F**). Given that both innate immunity hyperactivation and MB morphology phenotypes elicited by neuronal *Edis* depletion are suppressed in flies carrying mutations in *Relish*, our data strongly suggest the presence of additional *Edis* target/effect gene(s) beside AMPs that act downstream of Relish to regulate MB morphology. To search for these gene(s), we performed RNA-seq analysis using *Edis*-depleted and control brain tissues, from which we identified a total of 777 transcripts that displayed significant changes in RNA levels upon *Edis* knockdown (412 upregulated and 365 downregulated) (**S1 Table** and **Fig 4G**). Notably, several AMP genes (*i*.*e*., *CecA1*, *CecA2*, *CecB*, *CecC*, *DptA*, *AttC*, *Mtk*, *Dro* and *Drs*) were among the group of upregulated genes (**Fig 4G**), thereby validating our approach. The significantly changed genes could be grouped into 16 functional categories based on their predicted/validated roles using Gene Ontology (GO) (**Fig 4H**). Among these groups of genes, four are related to immune responses (**Fig 4H**), in addition to genes implicated in nervous system development. As flies missing neuronal *Edis* display profound neurodevelopmental phenotypes, we selected 17 neurodevelopment-related genes and performed RT-qPCR to examine their expression level in *shEdis* brain tissues. We note that not all of the 17 genes have scored in our RNA-seq analysis. Among the genes analyzed, *castor* was the most significantly activated in *Edis* knockdown brain (**Fig 4I**). *Castor* encodes a transcription factor that is expressed in late stages of embryonic neuroblast lineages, and has been shown to be involved in MB development [56], raising an intriguing possibility that dysregulation of *castor* expression by *Edis* depletion might be (at least partially) responsible for the MB morphology phenotypes in the CNS.

### *Castor* functions downstream of *Edis* in MB neuronal development

**Fig 5:**
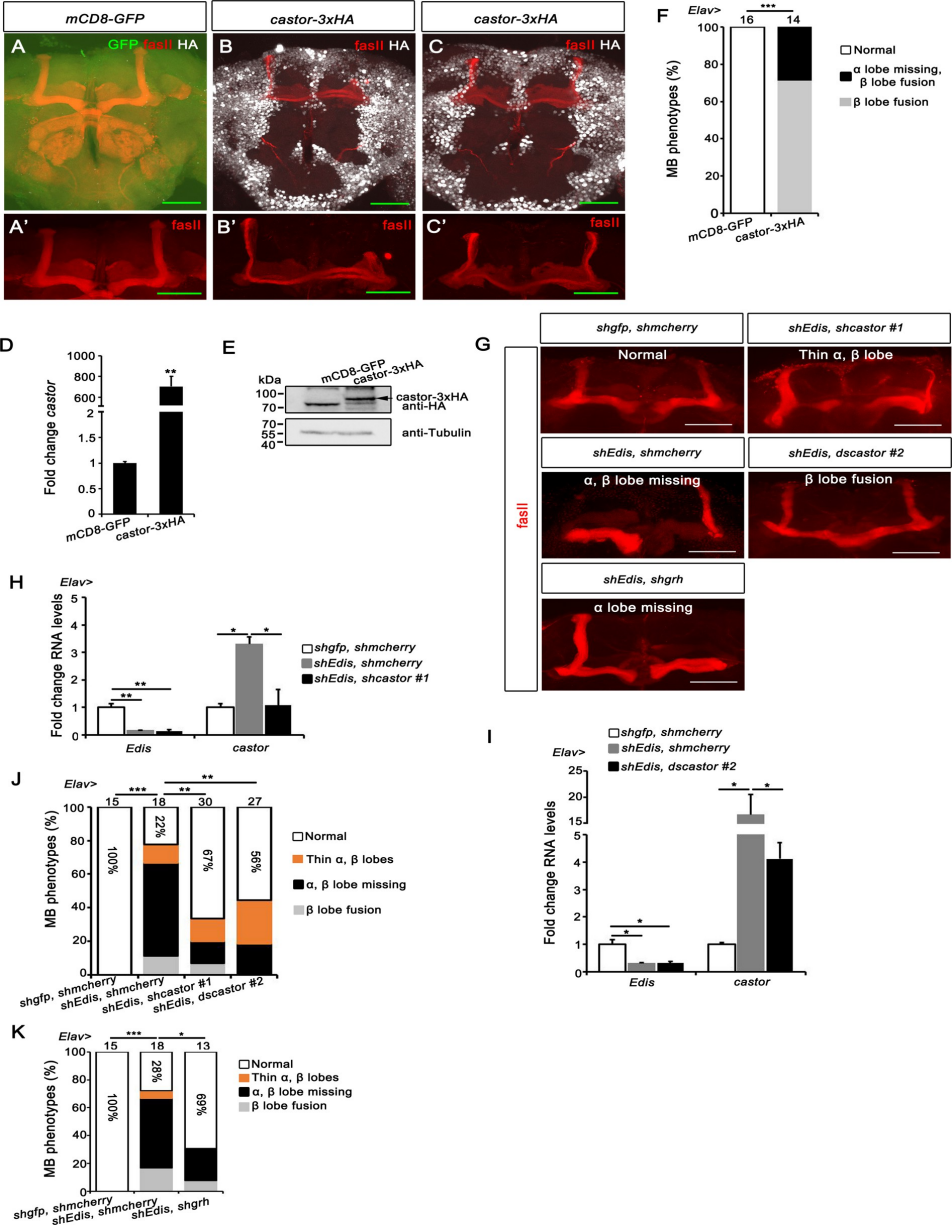
*Castor* functions downstream of *Edis* in MB neurodevelopment. (**A**-**C’**) Overexpression of *castor* in neurons led to MB morphology defects. *UAS-castor*-*3XHA* or *UAS-mCD8GFP* (control) flies were crossed to *Elav-Gal4* flies. Brains of progeny of indicated genotypes were stained by anti-HA and anti-FasII antibodies. **A’**, **B’** and **C’** show images of MB area in **A**, **B** and **C**, respectively. The scale bar indicates 50 μm. (**D-E**) Levels of *castor* mRNA and castor-HA protein were measured by real-time PCR (**D**) and immunoblot (**E**), respectively and compared to control (n = 3). (**F**) MB morphology phenotypes of indicated genotypes in **A**-**C’** were quantified. Chi-squared test was employed in statistical analysis. Sample numbers of each genotype are shown. (**G-J**) Reducing levels of *castor* expression rescues the MB morphology phenotypes of *Edis* knockdown brains. Various combinations of *UAS-shgfp*, *UAS-shEdis*, *UAS-shmcherry*, *UAS-shcastor* and *UAS-dscastor* transgenes were crossed with *Elav-Gal4* flies. Representative MB images of progeny of the indicated genotypes are shown in **G**. The scale bar indicates 50 μm. Levels of *Edis* and *castor* transcripts in fly brains of the indicated genotypes are measured by real-time PCR in **H** and **I** (student t test, n = 3). Quantification of MB morphology phenotypes with indicated genotypes in **H** and **I** is shown in **J**. Chi-squared test was employed in statistical analysis. Sample numbers and percentages of samples showing normal MB morphology in each genotype are shown. (**K**) Depletion of *grh* rescues the MB morphology defects in *Edis*-depleted neurons. Various combinations of *UAS-shgfp*, *UAS-shmcherry*, *UAS-shEdis* and *UAS-shgrh* transgenes were crossed with *Elav-Gal4* flies. Quantification of MB morphology phenotypes with indicated genotypes is shown. Chi-squared test was employed in statistical analysis. Sample numbers and percentages of samples showing normal MB morphology in each genotype are shown.

Given our findings showing that the expression level of *castor* was dramatically increased in *Edis*-depleted CNS (**Fig 4I**), we next tested whether *castor* overexpression can lead to the MB morphology phenotypes similar to those observed in *Edis* knockdown animals. *Elav-Gal4* was employed to drive *castor-3xHA* or *mCD8GFP* (control) overexpression in the CNS (**Fig 5A–5C’**), as confirmed by measuring both RNA (**Fig 5D**) and protein levels of *castor-3xHA* expression (**Fig 5B, 5C** and **5E**) in the brain tissue. Compared with control samples, overexpression of *caster* in the CNS indeed resulted in strong MB morphology defects: including β lobe fusion (71%) and missing α lobe accompanied with β lobe fusion (29%) (**Fig 5A’–5C’** and **5F**). Similarly, when UAS-*castor-3xHA* was specifically expressed using the MB driver *ok107-Gal4*, 67% of brains display MB morphology defects (**S3A and S3B Fig**). These data demonstrate that overexpression of *castor* compromises MB development.

Next, we examined whether *castor* is important for *Edis* function by testing whether the MB morphology phenotype in *Edis*–depleted neurons could be suppressed by down regulation of *castor*. We employed two independent *castor* RNAi lines to minimize off-target effects. Both RNAi lines led to a dramatic decrease in levels of *castor* transcript in *Edis* knockdown brains (**Fig 5H and 5I**). Importantly, the MB morphology phenotype in *Edis*–depleted brains was partially rescued (**Fig 5J**, >50% normal MB morphology in *castor* knockdown samples vs. 22% in controls). Similarly, the short lifespan and mobility defects were rescued as well (**S3C and S3D Fig**). *Castor* is among the temporal transcription factors expressed in the embryonic neuroblast lineage, and promotes the expression of a downstream factor, *grainyhead* (*grh*) [57, 58]. Consistently, *grh* was also upregulated in *Edis* knockdown neurons (**S4A Fig**). Furthermore, overexpression of *grh* in MB neurons using *ok107-Gal4* also resulted in defective MB morphology: β lobe fusion (80%), missing β lobe (13%) and missing α lobe accompanied by β lobe fusion (7%) (**S4B Fig**). More importantly, the MB morphology phenotype in *Edis*–depleted brains could also be partially rescued upon knocking down *grh* expression (**Figs S4C** and **5K**, 69% normal MB morphology in *grh* knockdown samples vs. 28% in controls). Taken together, these data demonstrate that *castor* plays an important role downstream of *Edis* in regulating MB development in the CNS.

### Relish regulates *castor* transcription

**Fig 6:**
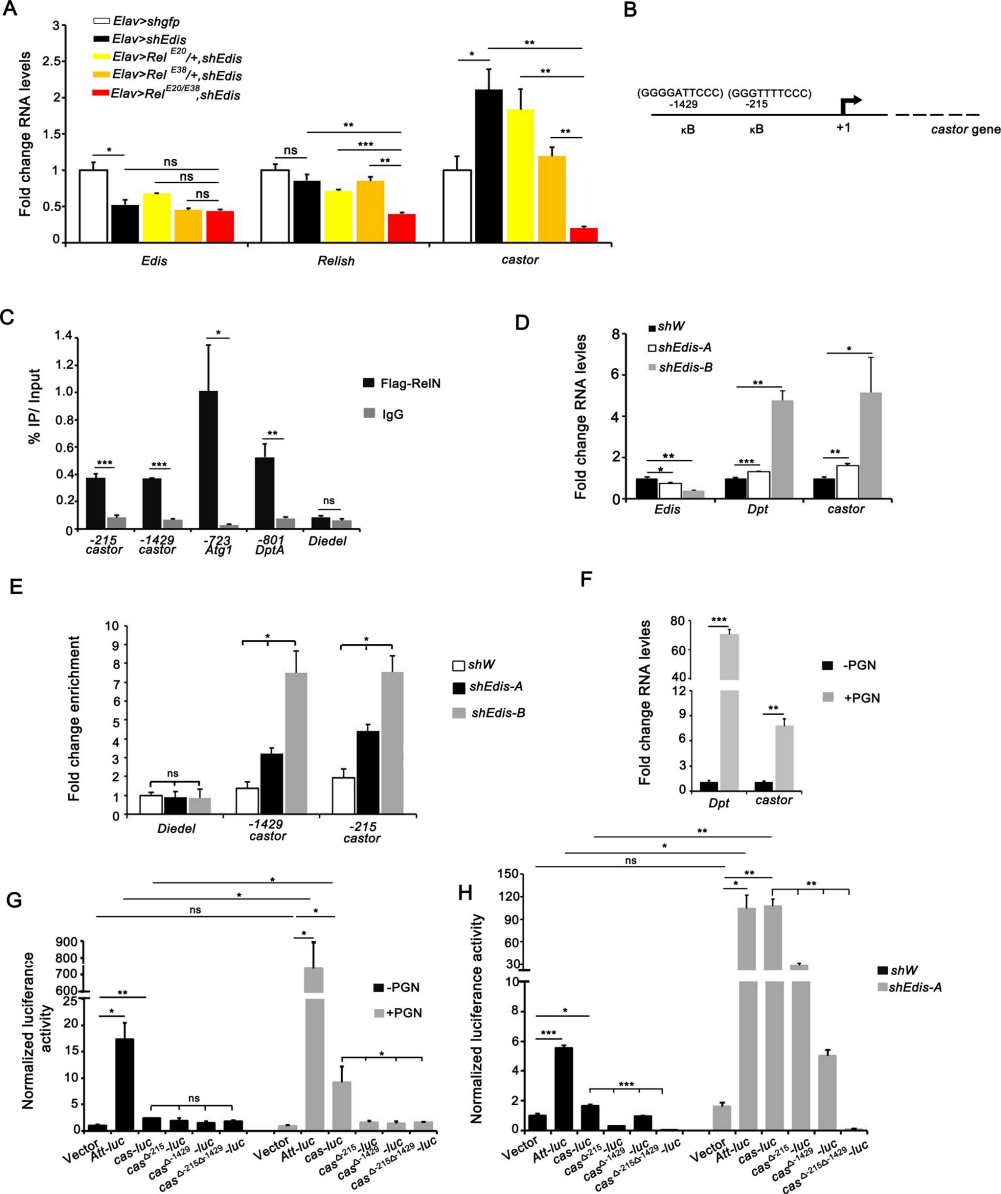
Relish regulates *castor* transcription. (**A**) Levels of *Edis*, *Relish*, and *castor* transcripts were measured in *Elav>shgfp* or *Elav>shEdis* flies in wildtype, *Relish* heterozygous (*Rel*^*E20*^/+ or *Rel*^*E38*^*/+*) or homozygous (*Rel*^*E20/E38*^) mutant background (n = 3). Levels of *castor* mRNA in *Elav>shEdis* flies are significantly lower in *Relish* homozygous (*Rel*^*E20/E38*^) compared with *Relish* heterozygous or wildtype background. (**B**) A diagram showing a pair of candidate Relish-binding sites on the *castor* promoter. (**C**) *UAS-Flag-RelN* was expressed in neurons using *Elav-Gal4*. Chromatin immunoprecipitation (ChIP) was performed in adult brain samples using anti-Flag antibody or IgG (control). Purified DNA was analyzed by using primer pairs amplifying the DNA fragments of *castor* promoter regions encompassing candidate Relish-binding sites. Two positive controls (position -723 of *Atg1* promoter and position -801 of *DptA* promoter) and negative control (*Diedel*) are shown (n = 2–5). (**D**) Levels of *Edis*, *Dpt*, and *castor* transcripts were measured in S2 cells stably transfected with *shW* (control), *shEdis*-*A*, or *shEdis-B* (n = 3–4). (**E**) Flag-Relish expression plasmid was introduced to S2 cells stably transfected with *shW* (control), *shEdis*-*A*, or *shEdis-B*. ChIP analysis was performed as in **C** (n = 2). (**F**) Levels of *Dpt* and *castor* transcripts were measured in S2 cells with or without PGN treatment (n = 3). (**G**) S2 cells were transfected with various luciferase reporter constructs in which the firefly luciferase reporter gene was placed downstream of wildtype or mutant *castor* promoter DNA fragments (missing one or both Relish-binding sites), together with the *pActin-Renilla* reporter (as a control for transfection efficiency). Cells were first treated with 20-HE and subsequently with PGN or left untreated. *Att-luc* and empty vector served as positive and negative controls, respectively. Normalized reporter activity is shown. Upon PGN treatment, both *Att-luc* and *cas-luc* were activated compared with controls. Reporter constructs missing either or both Relish-binding sites failed to respond to PGN treatment (n = 3). (**H**) The same set of reporter constructs as in **G** were introduced into S2 cells stably transfected with *shW* (control) or *shEdis*-*A*. Reporter activity was measured (n = 3).

*Edis* encodes a functional protein Edis-p that compromises proteolytic processing/activation of the immune transcription factor Relish, and inactivation of *Relish* in *Edis*-depleted neurons suppresses the innate immunity hyperactivation phenotype and rescues the neuronal developmental defects elicited by *Edis* depletion (**Fig 3**) [39]. Results of genome-wide Relish ChIP-seq analysis from ModENCODE (https://epic.gs.washington.edu/modERN/faces/index.xhtml;jsessionid=wJaqbmimlEFsQ3UYaWSPTJqytrJLTkEs9oogmBBc.epic) indicated that Relish binds to the *castor* promoter region (**S5A Fig**), suggesting that Relish may directly regulate *castor* transcription in *Edis-*depleted neurons. To explore this possibility, we introduced *Relish* mutant alleles into *Elav>shEdis* animals and measured the impact on the *castor* RNA levels. Indeed, upregulation of *castor* expression in *Elav>shEdis* brains was abrogated in *Relish* null mutant background (*Rel*^*E20*^*/Rel*^*E38*^) compared with *Relish* wildtype and heterozygous backgrounds (*Rel*^*E20*^/+ or *Rel*^*E38*^/+) (**Fig 6A**), supporting the notion that *Relish* is required for the upregulation of *castor* expression in *Edis*–depleted neurons.

We then searched for candidate Relish binding sites in the *caster* promoter based on conserved NF-κB-binding sequence [59]. We identified two candidate Relish binding sites upstream of the *castor* transcriptional start site (**Fig 6B**). To validate Relish occupancy at these sites, we performed chromatin immunoprecipitation (ChIP)-qPCR using dissected fly brains. Given that overexpression of full-length *Relish* in neurons driven by *Elav-Gal4* causes lethality, and that the antibody against endogenous Relish is not suitable for immunoprecipitation, we chose the *UAS-Flag-RelN* transgene that encodes the active form of Relish [60]. Interestingly, overexpression of *Flag-RelN* using the MB neuron-specific *ok107-Gal4* driver can lead to defects in MB morphology: only 11% of *ok107-Gal4>Flag-RelN* brains displayed normal MB morphology, whereas the remaining 89% showed MB morphology defects (**S5B and S5C Fig**). ChIP analysis of *Flag-RelN* expressing flies revealed that RelN binds to both sites of the *castor* promoter. The binding was specific as we detected high affinity binding of RelN to the promoters of two known *Relish* target genes (*Atg1* and *DptA*) [60]. No obvious binding was detected for the negative control, *Diedel* (**Fig 6C**). These data demonstrate that Relish binds to the *castor* promoter.

To further explore the role of Relish in regulating *castor* expression, we tested the expression of *castor* gene in response to *Edis* depletion in cultured cells. Consistent with our recent study [39], we detected an increase in *Dpt* RNA levels upon *Edis* depletion in S2 cells. Importantly, levels of *castor* were also similarly higher in *Edis* knockdown cells than in control cells (**Fig 6D**). These results are consistent with elevated *castor* expression detected in *Elav>shEdis* brains (**Fig 4I**). In addition, we also detected an increase in Relish occupancy on *castor* promoter upon *Edis* depletion in S2 cells (**Fig 6E**). To investigate the functional relevance of Relish binding to the *castor* promoter, we first examined whether *castor* gene expression can be induced by PGN treatment, a potent activator of Relish. Indeed, levels of *castor* mRNA were significantly increased in S2 cells treated with PGN, similar to the known Relish target gene *Dpt* (**Fig 6F**). Next, we generated reporter constructs in which the luciferase reporter gene was placed downstream of wildtype or mutant *castor* promoter lacking either or both Relish-binding sites, and then examined whether the reporter gene activity responds to PGN treatment. As a positive control, the *Att-luc* reporter, which is driven by the AMP gene *attacin* promoter, is activated by PGN treatment. Importantly, wildtype, but not mutant *castor* promoter constructs that lack either or both Relish binding sites, responded to PGN treatment (**Fig 6G**). Furthermore, we conducted similar luciferase reporter assays in *Edis*-depleted S2 cells. Both *att-luc* reporter and *cas-luc* displayed basal expression in the control cell line, but became dramatically activated upon *Edis* depletion (**Fig 6H**). Importantly mutant *castor* promoter constructs failed to respond to *Edis* depletion in the same assay (**Fig 6H**). We conclude that Relish binds to the *castor* promoter and regulates *castor* transcription, and that Relish binding is critically required for *castor* activation in response to PGN treatment or *Edis* depletion.

## Discussion

In a recent study we show that the circRNA *Edis* compromises innate immunity signaling and regulates neuronal development in *Drosophila* [39]. However, the detailed molecular mechanism underlying the role of *Edis* in neuronal development remains unclear. In this study, we report that *Edis* is primarily enriched in neurons in the brain, and its depletion causes defective axonal projection and abnormal MB morphology. Furthermore, our orthogonal genetic analysis using various developmental stage-specific Gal4 drivers and geneswitch system reveal that depletion of *Edis* either in neuroblasts or immature neurons (GMCs), but not in fully differentiated neurons, leads to MB developmental defects, therefore uncovering a crucial role of *Edis* during select stages of neuronal development. We note that while in this study we have been focusing on the mushroom body phenotypes primarily because these defects are easy to detect, it does not imply that the effect is specific to the mushroom body. In fact, we have uncovered additional neurodevelopmental defects in various neural structures in *Edis* knockdown animals (e.g. giant fiber, ommatidia and neuromuscular junction) [39]. Thus while the mushroom body defects result from cell-autonomous loss of *Edis* in MB neurons, *Edis* may be required in many other types of neurons as well.

*Relish* encodes a transcription factor with well-established roles in regulating innate immunity signaling. In addition, our analysis revealed mild MB morphology phenotypes in *Relish* null mutant animals (**Fig 3B**), consistent with recent reports that have implicated *Relish* in neurodevelopment [54,55]. We found that both the innate immunity hyperactivation and defective MB morphology phenotypes of *Edis* knockdown animals can be suppressed by either mutations or depletion of *Relish*. Thus our findings are in line with the notion that in *Drosophila* a small number of transcription factors are often re-used to regulate myriad biological processes. In this case, Relish is involved in the regulation of both innate immunity and neurodevelopment.

**Fig 7:**
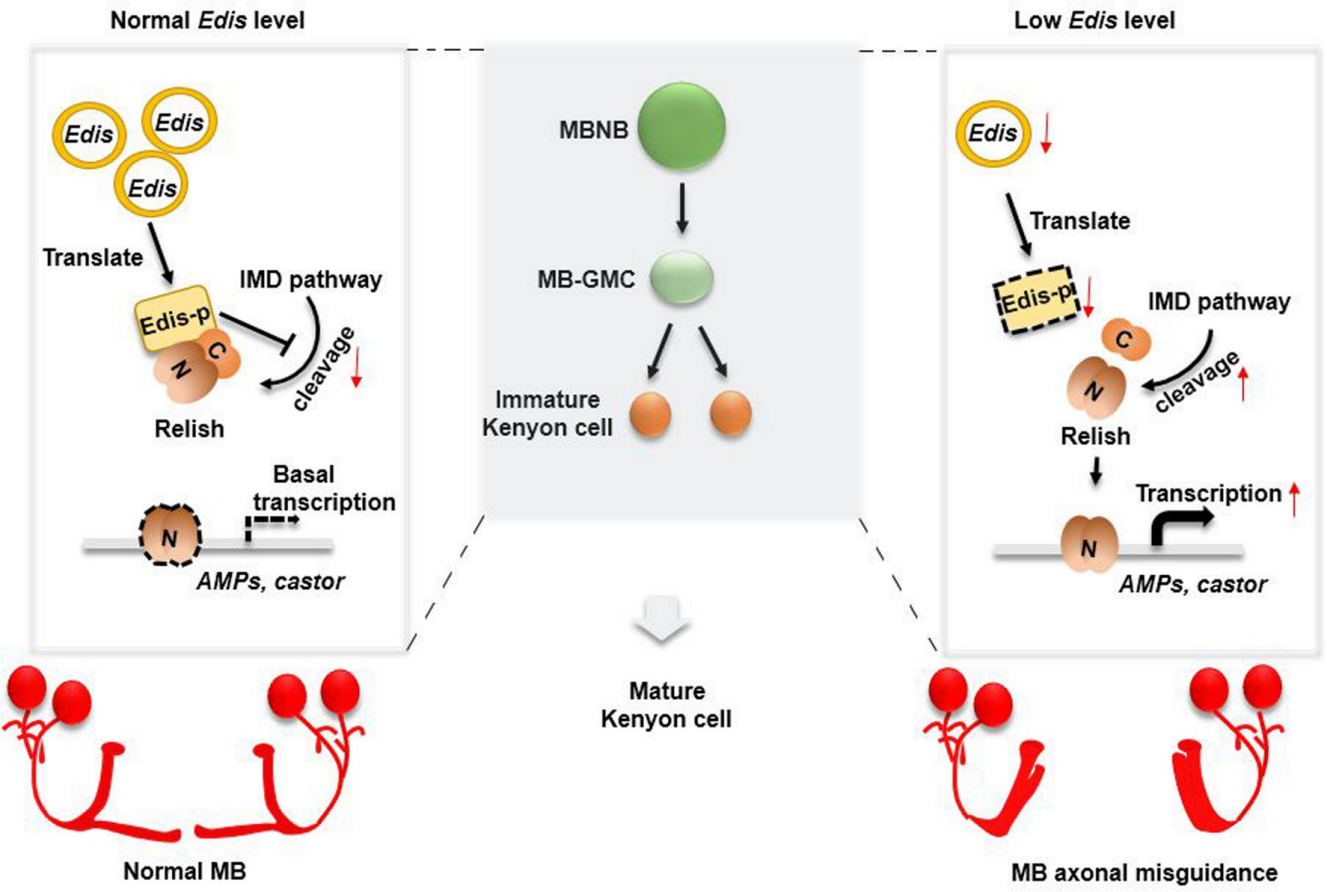
A diagram depicting the role of *Edis* in regulating neurodevelopment. With normal levels of *Edis* expression in *Drosophila* MB neuron precursors, *Edis* is translated into the Edis-p protein, which interacts with the immune transcription factor Relish and inhibits endoproteolytic processing/activation of Relish. Upon *Edis* depletion, Relish cleavage/activation is enhanced, which in turn promotes the transcription of both the AMP genes and *castor*, leading to MB neurodevelopmental defects.

Our study adds to a growing body of evidence supporting an intimate connection between dysregulation of immunity signaling and neurodevelopment. For example, it has been reported that ectopic expression of individual AMP genes or bacterial infection of the *Drosophila* brain is sufficient to cause brain damage [38]. Using MB morphology as an *in vivo* readout in an animal model, we show that forced expression of individual AMP genes in neurons indeed leads to abnormal MB morphology, although the defects are much milder than those elicited by neuronal *Edis* depletion (**Fig 4A–4F**). These findings suggest that besides AMPs, there are additional effector/target genes that operate downstream of *Edis* in regulating neurodevelopment. Indeed, we find that the neuronal transcriptional factor *castor* is significantly upregulated upon *Edis* depletion, and further establish *castor* as an important target/effector gene that acts downstream of *Edis* to regulate MB neuronal development. Specifically, we show that *castor* overexpression phenocopies *Edis* depletion in neurons, and that *castor* knockdown rescues the neurodevelopmental phenotype in *Edis*-depleted neurons. Lastly, our analyses reveal that upon *Edis* depletion, the immune transcription factor Relish binds to the *castor* promoter and directly upregulates *castor* transcription both in neurons and in cultured S2 cells. Thus, we propose that a circular RNA *Edis*-Relish-*castor* axis regulates neuronal development in *Drosophila melanogaster*, particularly in MB neurons (**Fig 7**).

We note that knocking down *castor* only partially rescues the MB neurodevelopmental defects in *Edis*-depleted animals, as there are still abnormal neurons (**Fig 5J**). It is possible that levels of *castor* are still not in the range of “right dosage” in these settings (**Fig 5I**). Additionally, we cannot exclude the possibility that there are additional factors besides *castor* that operate downstream of *Edis* in regulating neuronal development. Castor is among a group of temporal transcription factors in the neuronal lineage, including hunchback (hb), seven-up (svp), Krüppel (Kr), pdm (Flybase: nubbin and pdm2), and grainyhead (grh) [61–63]. Previous studies have implicated some of these factors in MB neurodevelopment. For example, it has been reported that *castor* and *svp* are required for generating small Chinmo^+^ neurons in many different lineages [64], and Chinmo controls the temporal identity of MB neurons [65]. Interestingly, we find that *grh* is upregulated in *Edis* knockdown brains (**S4A Fig**), and that overexpression of *grh* can also induce MB axonal misguidance (**S4B Fig**). Importantly, reducing *grh* expression levels partially rescues the MB morphology defects elicited by *Edis* depletion (**Fig 5K**). It is currently unclear whether any additional temporal transcription factors in the neuronal lineage may join *castor* and *grh* in mediating the neurodevelopmental defects observed in *Edis* depleted animals.

In light of the sequential/overlapping activities of a series of temporal transcription factors that regulate neuronal development, perhaps *Edis* and Relish could be part of such regulatory mechanism, which reinforces appropriate timing to stimulate *Castor* expression. Alternatively, *Edis* and Relish may be involved in regulating the generation of different types of Kenyon cells, in particular, late-born Kenyon cells (α/β neurons). In addition, circular RNAs are generally more stable than their linear siblings. Since downregulation of *Edis* leads to Relish activation, is *Edis* subjected to degradation and/or functional inhibition in order to relieve its inhibitory effects on Relish activation and *Castor* expression? Future studies are warranted to address these questions.

Here we demonstrate that the transcription factor Relish binds to *castor* promoter and regulates *castor* expression. We identify twin Relish-binding sites on the *castor* promoter, and show that both *cis*-regulatory elements are critically required for *castor* activation. Interestingly, *castor* can also be activated by PGN treatment, which potently induces the expression of innate immunity effector genes such as those encoding AMPs (**Fig 6F**). Thus our data uncover that the neurodevelopmental phenotypes elicited by *Edis* depletion is due to not only elevated levels of immune effectors such as AMPs, but also dysregulation of essential neuronal genes (e.g. *castor* and *grh*). The transcription factor Relish might serve as a crucial link that connects these two processes.

In summary, our study shows that the brain-enriched circular RNA *Edis* plays a crucial role in MB axonal guidance, and generates an animal model to investigate the role of circular RNAs in neuronal development and function. We identify and characterize the neuronal transcription factor *castor* as an effector/target gene of *Edis*, demonstrate that *castor* is transcriptionally regulated by Relish, and establish the function of *Edis*-Relish-*castor* axis in regulating neuronal development. Taken together, our study identifies a molecular link between innate immunity and neuronal development, broadens the spectrum of target genes that are transcriptionally regulated by Relish, and suggests a key role of Relish in regulating myriad biological processes including immunity, neurodevelopment and autophagy.

## Materials and Methods

### Statistical analyses

All statistical analyses in this manuscript were performed using biological replicates and the sample number (n) is shown for each dataset in the corresponding legend. Most analyses were performed using the two-tailed unpaired student *t*-test, except for lifespan experiments, which involved the log-rank test, and MB morphology experiments, which involved Chi-squared test. The data are presented as mean values + standard errors of the mean (SEM). A p value <0.05 was considered statistically significant. * *p*<0.05; ** *p*<0.01; *** *p*<0.001.

### DNA constructs and antibodies

To generate the luciferase reporter construct pGL3-cas-luc, the DNA fragment encompassing the *castor* promoter region (-1~-1924) was amplified from the *Drosophila* genomic DNA by PCR and inserted into the pGL3 vector using the Kpn I and Hind III restriction sites. Subsequently, based on the pGL3-cas-luc vector, three mutant constructs lacking either (pGL3-cas^Δ-215^-luc and pGL3-cas^Δ-1429^-luc) or both (pGL3-cas^Δ-215Δ-1429^-luc) Relish binding sites were generated using phusion site directed mutagenesis kit (Thermo Fisher, F541). To generate transgenic expression constructs for *DptA*, *Drs*, *Dro*, and *Def*, DNA fragments encompassing the ORF of these genes were amplified from the *Drosophila* cDNA by PCR and inserted into the pUAST vector using EcoR I and Xho I restriction sites. All constructs were verified by sequencing.

Antibodies used: mouse anti-Fas II (DSHB, 1D4) (IF 1:10); rabbit anti-GFP (Thermo Fisher, A11122) (IF 1:500); rabbit anti-HA (Cell Signaling, C29F4) (IF 1:400, WB 1:1000); M2 monoclonal mouse anti-Flag antibody (Sigma, F-3165) (WB 1:3000) and normal rabbit IgG (Millipore,12–370). Secondary antibodies: Alexa Fluor 488-conjugated anti-mouse IgG (Invitrogen, A21202); Alexa Fluor 488-conjugated anti-rabbit IgG (Invitrogen, A21206); Alexa Fluor 594-conjugated anti-mouse IgG (Invitrogen, A21203); Alexa Fluor 647-conjugated anti-rabbit IgG (Invitrogen, A31573) and goat anti-rabbit IgG antibody HRP conjugate (Millipore, 12–348).

### Fly genetics

Fly stocks are maintained on a standard fly food (Nutri-Fly, molasses formulation) and kept at 25°C. The genotypes of the fly stocks employed in this study were listed in the supplementary materials and methods.

### Cell culture and transfection

*Drosophila* S2 cells were cultured at 25°C in Schneider insect cell culture medium (Sigma-Aldrich, S0146) and supplemented with 10% fetal bovine serum (HyClone, SH30071.03) and 1% penicillin-streptomycin (Gibco, 15140122). For luciferase reporter assay, transfections were performed in a 24-well format by following the calcium phosphate protocol using 2.5M CaCl_2_ and 2XHEPES buffered saline.

### Immunofluorescence

For *Drosophila* mushroom body neuron fiber staining, adult fly brains were dissected, stained, and imaged as described [66]. Briefly, adult fly heads of 3–5 days were dissected in PTN buffer (0.1 M Sodium Phosphate Buffer, pH 7.2, 0.1% Triton X-100) and fixed with 4% paraformaldehyde (Alfa Aesar, 43368) for 20 minutes at room temperature, followed by rinsing three times in PTN buffer. Samples were blocked with 5% BSA at room temperature for 30 minutes and incubated with primary antibody at 4°C for 48 hours, followed by washing three times with PTN buffer. Samples were then incubated with secondary antibody at 4°C overnight and washed three times as indicated above. Subsequently samples were mounted in 80% glycerol and imaged using a Nikon confocal microscope (Nikon A1, Tokyo, Japan).

### Paraffin embedded sections and RNA fluorescence in situ hybridization

To prepare paraffin-embedded sections of *Drosophila*, dissected adult fly brains were fixed in 4% paraformaldehyde at room temperature for 24 hours, and washed two times with PBS. Samples were then dehydrated by incubating with increasing concentrations of ethanol (40%, 70%, and 100%) at room temperature, followed by incubation in ethanol/xylene solution (1:1) for 10 min. Samples were subsequently incubated in the following solutions at 60–65°C, xylene (30 min), xylene/paraffin (1:1, 30 min), paraffin (4 times, each time for ~1 hour). The sample container was then filled with liquid paraffin and rested at room temperature until the paraffin is solidified. Paraffin embedded samples were sectioned to a thickness of 5 ± 1 μm. For RNA fluorescence in situ hybridization (FISH), two probes were designed by Thermo Fisher: the 20-oligonucleotide *Edis* probe is complementary to the “back-spliced exon junction” of the circRNA *Edis*, whereas the control RNA probe is complementary to the *Choline acetyltransferase* (*ChAT*) mRNA. and RNA FISH was performed using the ViewRNA ISH Tissue 2-Plex Assay Kit (Affymetrix, QVT0012).

### Real time RT-PCR

*Drosophila* heads or S2 cells were collected and total RNA was isolated with TRIzol (Invitrogen, 15596026). RNA samples were subsequently reverse transcribed using Superscript III (Invitrogen, 18080044) and random hexamer primers, and levels of circular and linear RNAs were measured by quantitative PCR. The Real-time RT-PCR analysis was performed using the SYBR Green PCR master mix (BioRad, 1725275). Relative mRNA levels were calculated by normalization against the endogenous the control *RpL32* mRNA.

### Immunoblot

*Drosophila* heads were collected and homogenized in lysis buffer (25 mM Tris-HCl, pH 7.4, 150 mM NaCl, 1 mM EDTA, 5% glycerol and complete protease inhibitors). Lysates were centrifuged at 2,000 g for 5 min. Then 2X SDS loading buffer was added into the supernatant. Proteins were separated by SDS-PAGE gel and transferred onto a PVDF (Millipore, IPVH00010) membrane. The membrane was blocked with 5% non-fat milk solution and incubated with primary antibody at 4°C overnight, and washed 3 times with TBST buffer (20 mM Tris-HCl, 150 mM NaCl, 0.1% Tween 20). The membrane was then incubated with HRP-conjugated secondary antibody for 1 h at room temperature. The membrane was subsequently washed 3 times with TBST buffer, incubated with ECL (Cyanagen Srl, XLS3-0020) reagents. Images were acquired using ChemiDoc (Bio-Rad).

### Chromatin immunoprecipitation

To perform ChIP assay using *Drosophila* S2 cells, we followed the protocol reported in our recent study [39]. As for ChIP assay using *Drosophila* brains, we followed the protocol reported by [67] with minor modifications. Briefly, adult fly brain samples were dissected in ice-cold PBS and fixed in 1 mL cross-linking solution (1.8% formaldehyde, 50 mM HEPES pH 8.0, 1 mM EDTA, 0.5 mM EGTA, 100 mM NaCl) at room temperature. The cross-linking solution was changed 3–4 times during fixation. Cross-linking is terminated by adding 125 mM glycine. Samples were washed in 1 ml buffer A (10 mM HEPES pH 7.6, 10 mM EDTA, 0.5 mM EGTA, 0.25% Triton X-100) for 10 min and subsequently in 1 mL buffer B (10 mM HEPES pH 7.6, 200 mM NaCl, 1 mM EDTA, 0.5 mM EGTA, 0.01% Triton X-100) for 10 min. Samples were homogenized in in 0.5 mL RIPA buffer (140 mM NaCl, 10 mM Tris-HCl pH 8.0, 1 mM EDTA, 1% Triton X-100, 0.1% SDS, 0.1% sodium deoxycholate, 1 mM PMSF, 0.5% N-Laurylsarcosine, complete protease inhibitor cocktail), and subsequently sonicated for 22 times for 30 seconds at “High” setting (Bioruptor 300, Diagenode). Lysate was centrifugated for 20 minutes at 4°C at a speed of 16000 g and diluted up to 7.2 mL with RIPA buffer. Ten microliters of diluted chromatin was saved as input control, and 240 μL of sonicated chromatin solution was diluted with 1 mL RIPA buffer for immunoprecipitation process. One hundred microliters of protein A sepharose CL-4B beads (GE healthcare, 17078001) or 40 μl of mouse M2 anti-Flag conjugated agarose beads (Sigma, A2220) were equilibrated in 1 ml RIPA buffer, incubated at 4°C for 1 h, and centrifuged at 4°C for 10 min at 16000 g. Equilibrated M2 anti-Flag conjugated agarose beads were resuspended with the chromatin sample, and protein A sepharose CL-4B beads resuspend with the same amount of chromatin with 2 μl IgG as control. Samples were incubated at 4°C overnight, centrifuged for 1 minute at 2000 g and supernatant was removed. Samples were then washed sequentially with the following buffers: RIPA buffer (5 times), and once with LiCl wash buffer (0.25 M LiCl, 1 mM EDTA, pH8.0, 0.5% NP-40, 0.5% Sodium Deoxycholate, 10 mM Tris-HCl, pH8.0). DNA was eluted with 100 μL elution buffer (1% SDS, 100 mM NaHCO3) by vortexing slowly for 30 minutes at 30°C. Samples were centrifuged for 1 min at 2000 g and the supernatant was transferred into a new tube. Four point eight microliters of 5 M NaCl and 2 μL RNase A (10 mg/mL) were added and samples were incubated at 65°C overnight. Subsequently 2 μL of proteinase K (20 mg/mL) was added and samples were incubated at 65°C for an additional hour. DNA was subsequently purified by phenol/chloroform extraction.

### Luciferase assay

Briefly, ~5×10^5^ of *shW*, *shEdis-A*, and *shEdis-B* stably transfected S2 cells or the parental S2 cells were seeded in 24-well plates the day before transfection. Subsequently, 500 ng empty pGL3 vector, *att-luc*, *cas-luc*, cas^Δ-215^-luc, cas^Δ-1429^-luc, or cas^Δ-215Δ-1429^-luc reporter constructs were transfected into these cells together with 20 ng of *actin-Renilla luciferase* plasmid. Two days post transfection, cells were treated with 250 μM copper for ~3–5 days, to achieve *Edis* knockdown. Transfected parental S2 cells were further treated with 1 μM 20-hydroxyecdysone (Sigma, H5142) for 24 hours and subsequently left untreated or treated with PGN for 6 hours. Cell suspensions were arrayed in 96-well plates and reporter activity was measured using the Dual-Glo luciferase assay system (Promega, E2920). For data processing, firefly/*Renilla* ratio was calculated and normalized against control samples.

### *Drosophila* lifespan and locomotor activity

For lifespan experiments, flies of the indicated genotypes were kept at 25°C (in multiple groups per genotype, 15 flies per group) and survival was monitored daily. To measure locomotor activity, we followed the protocol reported by Liu *et al* [68] with minor modifications. Briefly, flies in multiple groups of 15 were placed into Falcon culture tubes. Incubated at room temperature for 5 min and tapped to the bottom, and the percentage of flies that can climb over the 2-centimeter mark within 15 seconds was recorded.

### RNA-Seq data analysis

RNA-Seq datasets were generated from RNA samples extracted from *Elav>shEdis* and *Elav>shGFP* (control) fly heads. Each library was sequenced with paired-end 100 bp reads to a minimum depth of 40 million paired reads on an Illumina HiSeq 4000 sequencer (Illumina). The raw reads were aligned to the *Drosophila*. *melanogaster* reference genome by the HISAT2 aligner (v2.0.4) [69] with the default parameters. Ambiguous reads that mapped to more than one region in the genome and aligned reads with MAPQ score less than 10 were removed. The *Drosophila*. *melanogaster* reference genome (dm6) and corresponding RefSeq annotation (refFlat.txt.gz 28-May-2017) downloaded from UCSC were used as a reference genome for gene quantification. Gene quantification was performed using the Partek Genomics Suite (version 7.17, Partek), and the raw read counts and normalized read counts (reads per kilobase per million mapped reads [RPKM]) were obtained. Gene with poor read counts in all samples were excluded from further analysis. The differences in gene expression between knockdown and GFP control conditions were assessed using BioConductor edgeR package [70]. The resulting p-values were adjusted using the Benjamini and Hochberg method to control the false discovery rate. Genes with fold-change (FC) over two and p-value less than 0.05 were considered as significantly differentially expressed genes (DEGs). The sequencing data were deposited to Gene Expression Omnibus with the accession number GSE196213.

## Supporting information

S1 Fig *Edis* overexpression alone does not impact MB morphology. (**A-F’**) Fluorescence in situ hybridization (FISH) was employed to visualize *Edis* (red) in adult fly brains of control (*Elav>shgfp*) and *Edis* knockdown (*Elav>shEdis*) animals. Nuclei were marked by DAPI (blue). **A** and **B,** and **D** and **E** are split channels of **C** and **F**, respectively. **A’-C’** and **D’-F’** show high magnification images of boxed regions in **A-C** and **D-F**, respectively. The scale bars indicate 50 μm in **A-F**, and 10 μm in **A’-F’**. (**G**) *UAS-laccase 2-Edis* or *UAS-laccase 2 vector* (control) flies were crossed to *Elav-Gal4* flies and MB morphology was revealed by anti-FasII antibody staining. (**H**) MB morphology phenotypes of indicated genotypes were quantified. Chi-squared test was employed in statistical analysis. Sample numbers in each genotype are shown. (**I**) Levels of *Edis* were measured by real time PCR (n = 3).
(TIF)

S2 Fig AMP genes are upregulated upon neuronal *Edis* depletion. Levels mRNA encoding various AMPs (*DptA*, *Dro*, *Def*, *Drs*) in *Elav>shEdis* brain tissues and control samples were measured by quantitative PCR (n = 3).
(TIF)

S3 Fig *castor* is an effector/target gene of *Edis* in MB neurodevelopment. (**A**-**B**) Overexpression of *castor* in neurons led to MB morphology defects. *UAS-castor*-*3XHA* or *UAS-mCD8GFP* (control) flies were crossed to *ok107-Gal4* flies. Brains of progeny of indicated genotypes were stained by anti-FasII antibody. The scale bar indicates 50 μm. (**B**) MB morphology phenotypes of indicated genotypes were quantified. Chi-squared test was employed in statistical analysis. Sample numbers and percentages of samples showing normal MB morphology in each genotype are shown. (**C-D**) The short lifespan phenotype and mobility defects elicited by *Edis* depletion can be rescued by reducing *castor* expression. Various combinations of *UAS-shgfp*, *UAS-shEdis*, *UAS-shmcherry*, and *UAS-dscastor* transgenes were crossed with *Elav-Gal4* flies. Lifespan (**C**) and locomotor activity (**D**) of flies with indicated genotypes are shown.
(TIF)

S4 Fig *grh* is upregulated upon neuronal *Edis* depletion. (**A**) RNA levels of genes encoding a series of temporal transcription factors in fly brain samples were measured by real-time PCR and normalized to control samples. *grh* was among the upregulated genes in *Edis*-depleted fly brain (n = 6). (**B**) Overexpression of *grh* in the MB neurons led to MB morphology defects. *UAS-grh* or *UAS-mCD8GFP* (control) flies were crossed to *ok107-Gal4* flies. Brains of progeny of indicated genotypes were stained by anti-FasII antibody. MB morphology phenotypes of indicated genotypes were quantified. Chi-squared test was employed in statistical analysis. Sample numbers of each genotype are shown. (**C**) Various combinations of *UAS-shgfp*, *UAS-shmcherry*, *UAS-shEdis* and *UAS-shgrh* transgenes were crossed with *Elav-Gal4* flies. Levels of *Edis* and *grh* transcripts in flies of the indicated genotypes were measured by quantitative PCR (n = 3). (**D**) Levels of *Edis*, *Relish*, and *grh* transcripts were measured in control *Elav>shgfp* or *Elav>shEdis* animals in wildtype, *Relish* heterozygous (*Rel*^*E20*^/+ or *Rel*^*E38*^*/+*) or homozygous (*Rel*^*E20/E38*^) mutant background (n = 3). Levels of *grh* mRNA in *Elav>shEdis* flies are significantly lower in *Relish* homozygous (*Rel*^*E20/E38*^) compared with *Relish* heterozygous or wildtype background.
(TIF)

S5 Fig Relish binds to the promoter region of *castor* and induces MB morphology defects. (**A**) Integrative genomics viewer (IGV) plot of Rel-N-GFP ChIP sequence result showing the Relish peaks at the promoter region of *castor*. (**B-C**) RelN overexpression in MB neurons led to MB morphology defects. *UAS-Flag-RelN* or *UAS-mCD8GFP* (control) flies were crossed to *Elav-Gal4* flies. Brains of progeny of indicated genotypes were stained by anti-FasII antibody (**B**). The scale bar indicates 50 μm. MB morphology phenotypes of indicated genotypes were quantified in **C**. Chi-squared test was employed in statistical analysis. Sample numbers and percentages of samples showing normal MB morphology in each genotype are shown.
(TIF)

S1 Table Differentially expressed genes upon *Edis* knockdown in neurons. RNA-seq analysis was performed using RNA samples extracted from *Elav>shEdis* or *Elav>shGFP* (control) fly heads. Differential expressed genes (DEGs) in knockdown vs Control were defined using cut-off criteria of absolute fold change of > = 2.0 and p value of = < 0.05.
(XLSX)

S1 Data Numerical data underlying the figures. (XLSX)

S2 Data Images underlying the figures. (RAR)

S1 Information Sequence of oligonucleotides and genotypes of flies. (DOCX)
