# Supplementary figures and images for: A circular RNA Edis-Relish-castor axis regulates neuronal development in Drosophila
Source: PLoS Genet. 2022 Oct 27;18(10):e1010433. doi: 10.1371/journal.pgen.1010433 (PMC9612563; doi:10.1371/journal.pgen.1010433)

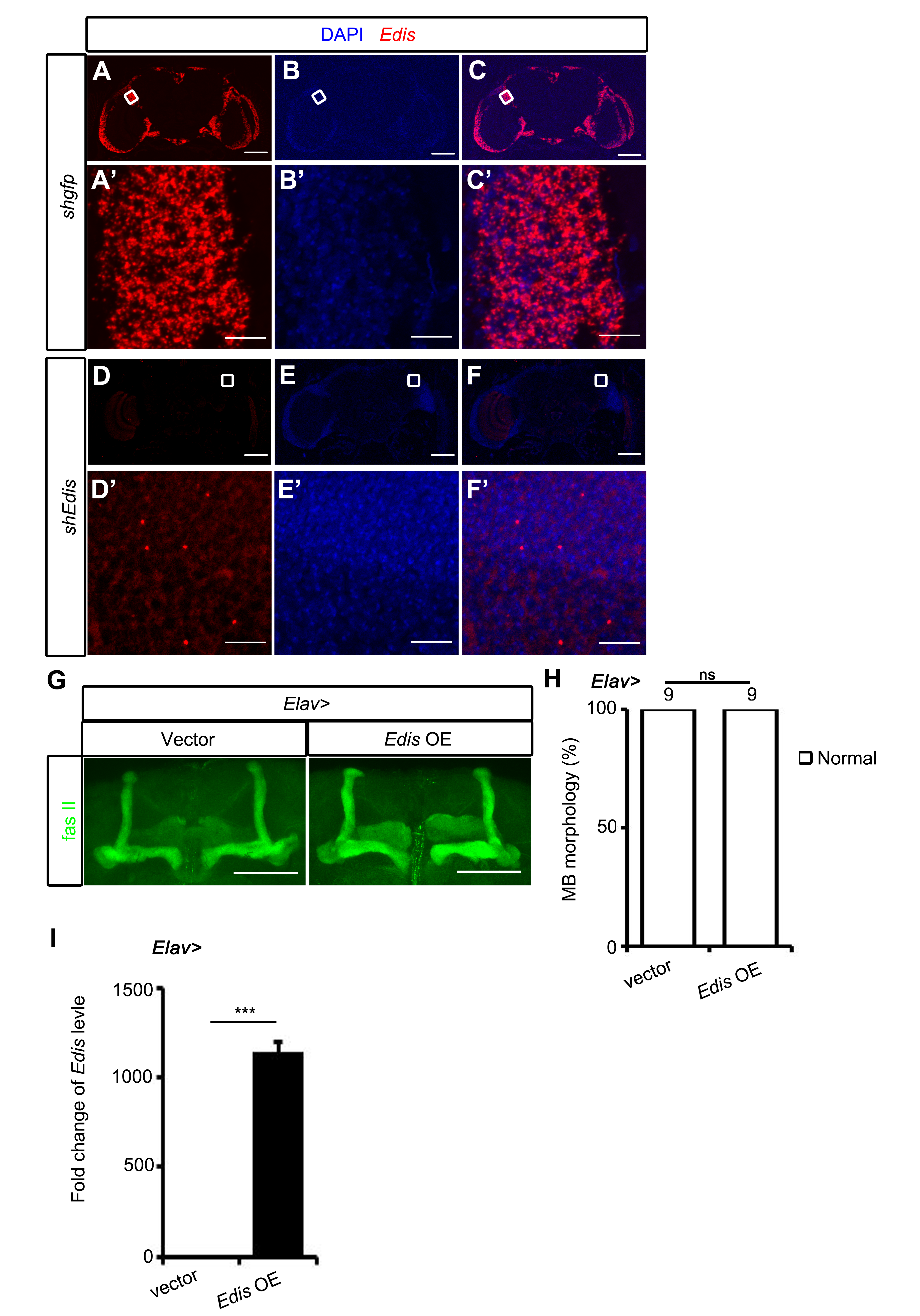

Supplement: S1 Fig — (A-F’) Fluorescence in situ hybridization (FISH) was employed to visualize Edis (red) in adult fly brains of control (Elav>shgfp) and Edis knockdown (Elav>shEdis) animals. Nuclei were marked by DAPI (blue). A and B, and D and E are split channels of C and F, respectively. A’-C’ and D’-F’ show high magnification images of boxed regions in A-C and D-F, respectively. The scale bars indicate 50 μm in A-F, and 10 μm in A’-F’. (G) UAS-laccase 2-Edis or UAS-laccase 2 vector (control) flies were crossed to Elav-Gal4 flies and MB morphology was revealed by anti-FasII antibody staining. (H) MB morphology phenotypes of indicated genotypes were quantified. Chi-squared test was employed in statistical analysis. Sample numbers in each genotype are shown. (I) Levels of Edis were measured by real time PCR (n = 3). (TIF) [file pgen.1010433.s001.tif]

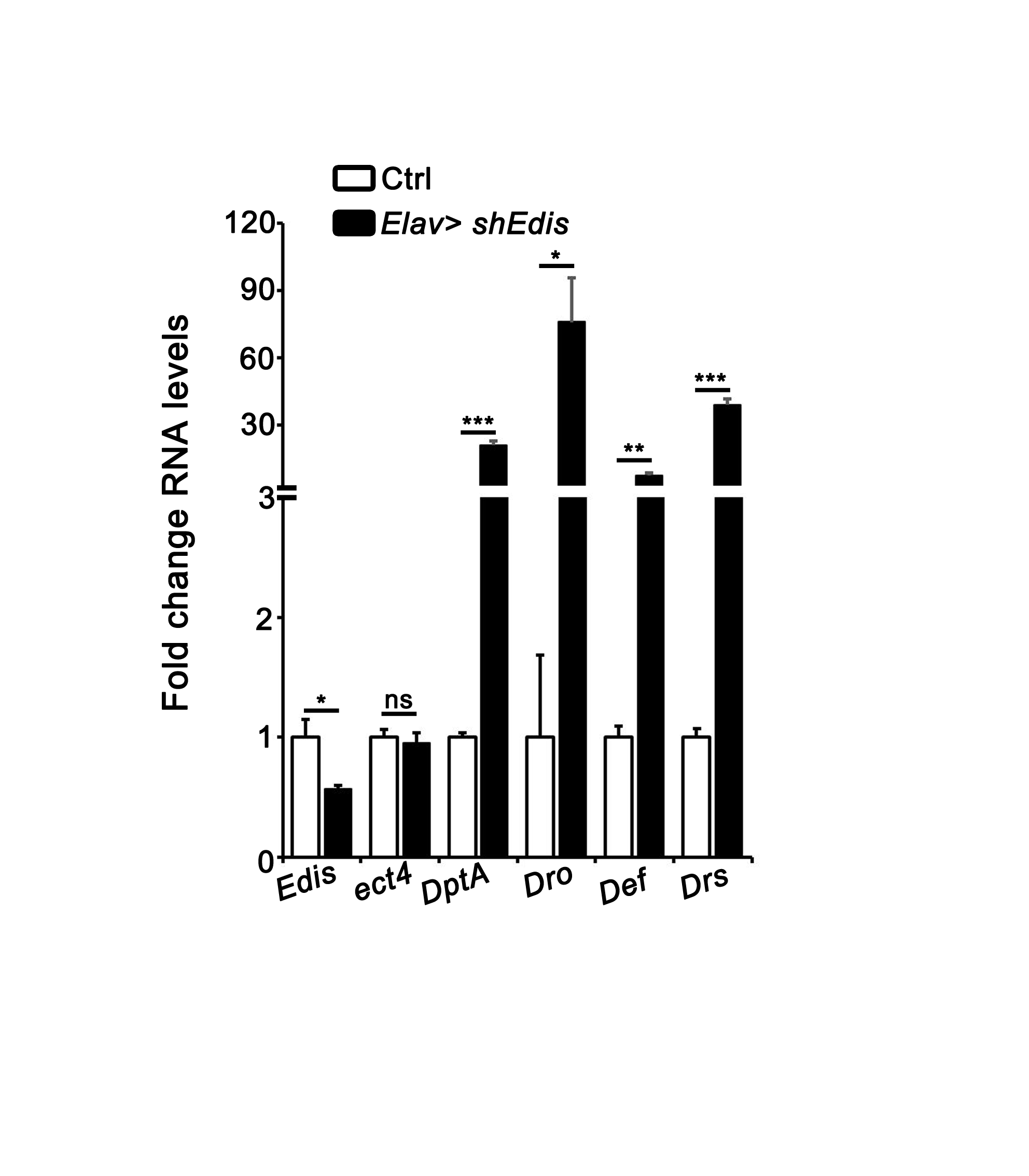

Supplement: S2 Fig — Levels mRNA encoding various AMPs (DptA, Dro, Def, Drs) in Elav>shEdis brain tissues and control samples were measured by quantitative PCR (n = 3). (TIF) [file pgen.1010433.s002.tif]

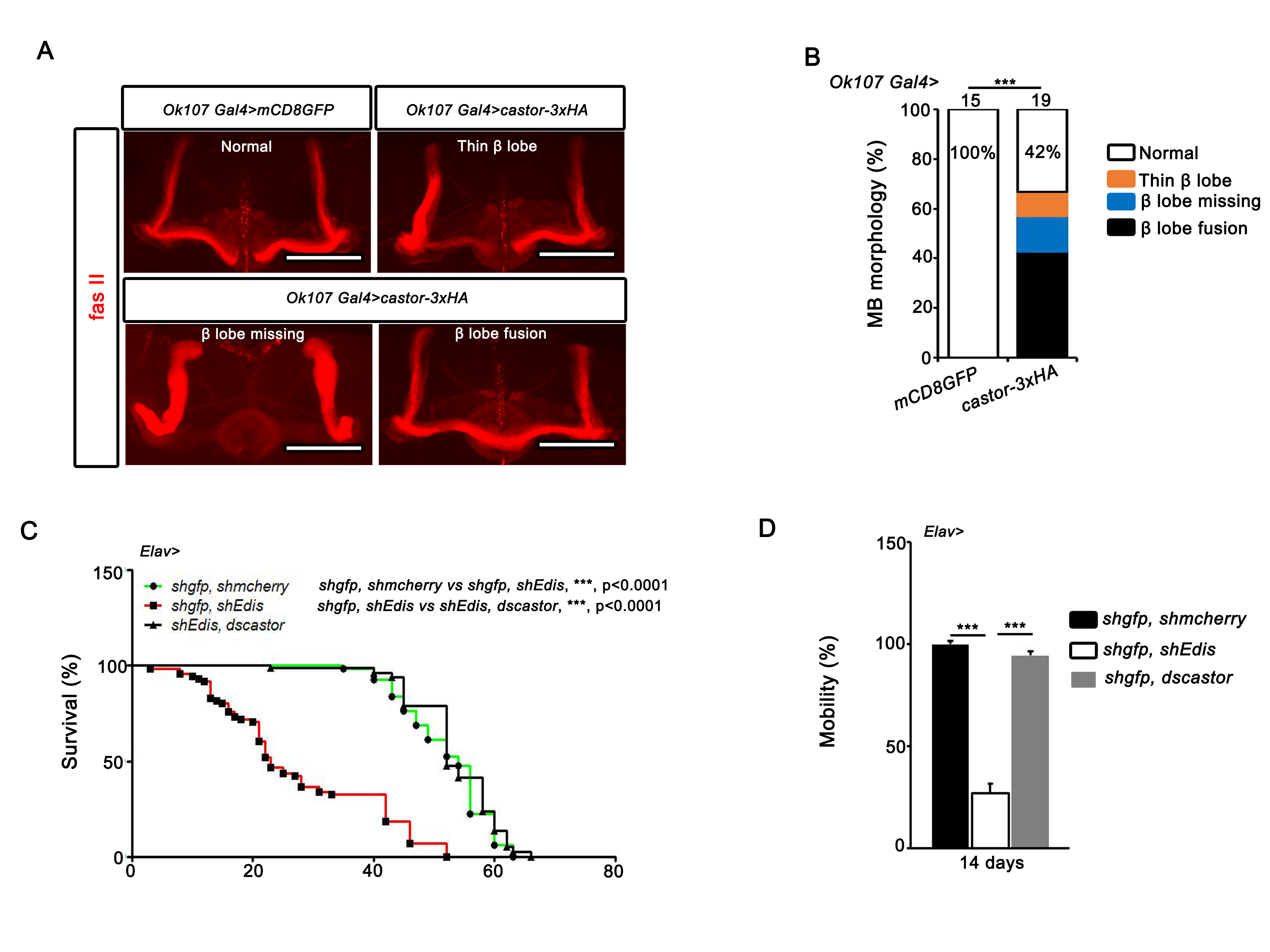

Supplement: S3 Fig — (A-B) Overexpression of castor in neurons led to MB morphology defects. UAS-castor-3XHA or UAS-mCD8GFP (control) flies were crossed to ok107-Gal4 flies. Brains of progeny of indicated genotypes were stained by anti-FasII antibody. The scale bar indicates 50 μm. (B) MB morphology phenotypes of indicated genotypes were quantified. Chi-squared test was employed in statistical analysis. Sample numbers and percentages of samples showing normal MB morphology in each genotype are shown. (C-D) The short lifespan phenotype and mobility defects elicited by Edis depletion can be rescued by reducing castor expression. Various combinations of UAS-shgfp, UAS-shEdis, UAS-shmcherry, and UAS-dscastor transgenes were crossed with Elav-Gal4 flies. Lifespan (C) and locomotor activity (D) of flies with indicated genotypes are shown. (TIF) [file pgen.1010433.s003.tif]

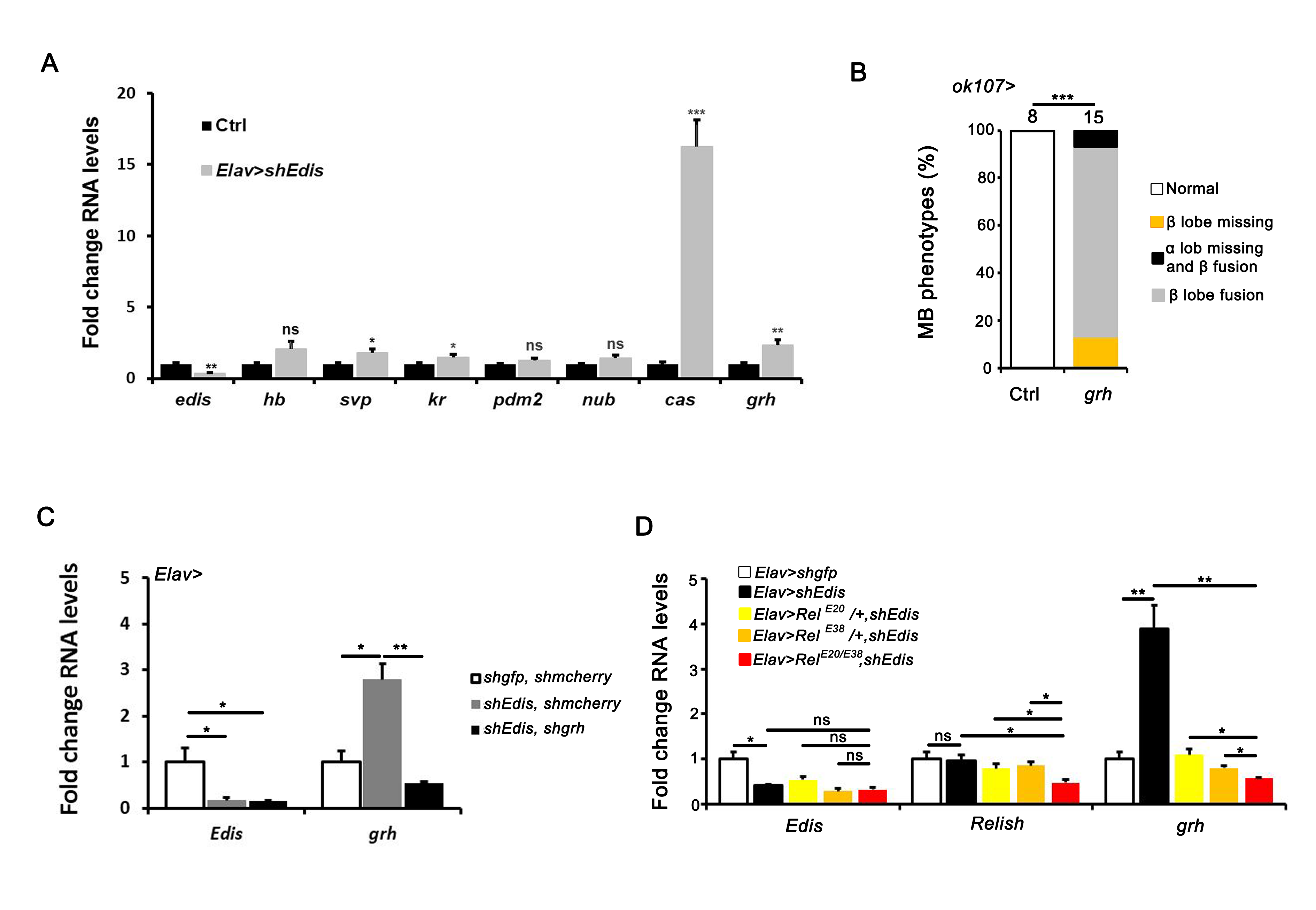

Supplement: S4 Fig — (A) RNA levels of genes encoding a series of temporal transcription factors in fly brain samples were measured by real-time PCR and normalized to control samples. grh was among the upregulated genes in Edis-depleted fly brain (n = 6). (B) Overexpression of grh in the MB neurons led to MB morphology defects. UAS-grh or UAS-mCD8GFP (control) flies were crossed to ok107-Gal4 flies. Brains of progeny of indicated genotypes were stained by anti-FasII antibody. MB morphology phenotypes of indicated genotypes were quantified. Chi-squared test was employed in statistical analysis. Sample numbers of each genotype are shown. (C) Various combinations of UAS-shgfp, UAS-shmcherry, UAS-shEdis and UAS-shgrh transgenes were crossed with Elav-Gal4 flies. Levels of Edis and grh transcripts in flies of the indicated genotypes were measured by quantitative PCR (n = 3). (D) Levels of Edis, Relish, and grh transcripts were measured in control Elav>shgfp or Elav>shEdis animals in wildtype, Relish heterozygous (RelE20/+ or RelE38/+) or homozygous (RelE20/E38) mutant background (n = 3). Levels of grh mRNA in Elav>shEdis flies are significantly lower in Relish homozygous (RelE20/E38) compared with Relish heterozygous or wildtype background. (TIF) [file pgen.1010433.s004.tif]

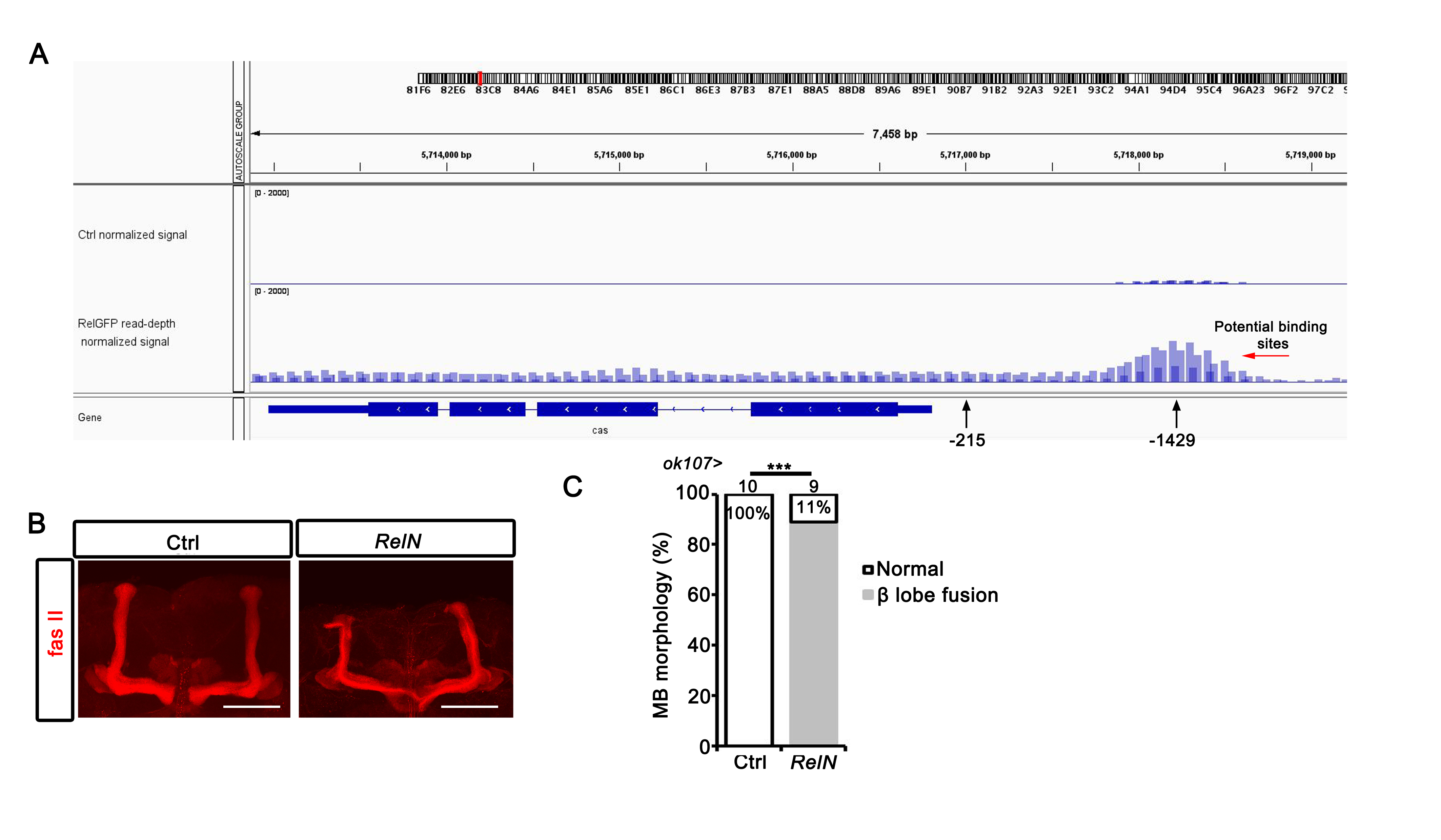

Supplement: S5 Fig — (A) Integrative genomics viewer (IGV) plot of Rel-N-GFP ChIP sequence result showing the Relish peaks at the promoter region of castor. (B-C) RelN overexpression in MB neurons led to MB morphology defects. UAS-Flag-RelN or UAS-mCD8GFP (control) flies were crossed to Elav-Gal4 flies. Brains of progeny of indicated genotypes were stained by anti-FasII antibody (B). The scale bar indicates 50 μm. MB morphology phenotypes of indicated genotypes were quantified in C. Chi-squared test was employed in statistical analysis. Sample numbers and percentages of samples showing normal MB morphology in each genotype are shown. (TIF) [file pgen.1010433.s005.tif]
