# Supplementary material for: A circular RNA Edis-Relish-castor axis regulates neuronal development in Drosophila
Source: PLoS Genet. 2022 Oct 27;18(10):e1010433. doi: 10.1371/journal.pgen.1010433 (PMC9612563; doi:10.1371/journal.pgen.1010433)
Supplement: S1 Information — (DOCX) [file pgen.1010433.s009.docx]

**Supplementary Information:**

**S1 Fig. *Edis* overexpression alone does not impact MB morphology.** (**A-F’**) Fluorescence in situ hybridization (FISH) was employed to visualize *Edis* (red) in adult fly brains of control (*Elav>shgfp*) and *Edis* knockdown (*Elav>shEdis*) animals. Nuclei were marked by DAPI (blue). **A** and **B,** and **D** and **E** are split channels of **C** and **F**, respectively. **A’-C’** and **D’-F’** show high magnification images of boxed regions in **A-C** and **D-F**, respectively. The scale bars indicate 50 µm in **A-F**, and 10 µm in **A’-F’**. (**G**) *UAS-laccase 2-Edis* or *UAS-laccase 2 vector* (control) flies were crossed to *Elav-Gal4* flies and MB morphology was revealed by anti-FasII antibody staining. (**H**) MB morphology phenotypes of indicated genotypes were quantified. Chi-squared test was employed in statistical analysis. Sample numbers in each genotype are shown. (**I**) Levels of *Edis* were measured by real time PCR (n=3).

**S2 Fig. AMP genes are upregulated upon neuronal *Edis* depletion.** Levels mRNA encoding various AMPs (*DptA, Dro, Def, Drs*) in *Elav>shEdis* brain tissues and control samples were measured by quantitative PCR (n=3).

**S3 Fig. *castor* is an effector/target gene of *Edis* in MB neurodevelopment.** (**A**-**B**) Overexpression of *castor* in neurons led to MB morphology defects. *UAS-castor*-*3XHA* or *UAS-mCD8GFP* (control) flies were crossed to *ok107-Gal4* flies. Brains of progeny of indicated genotypes were stained by anti-FasII antibody. The scale bar indicates 50 µm. (**B**) MB morphology phenotypes of indicated genotypes were quantified. Chi-squared test was employed in statistical analysis. Sample numbers and percentages of samples showing normal MB morphology in each genotype are shown. (**C-D**) The short lifespan phenotype and mobility defects elicited by *Edis* depletion can be rescued by reducing *castor* expression. Various combinations of *UAS-shgfp*, *UAS-shEdis*, *UAS-shmcherry*, and *UAS-dscastor* transgenes were crossed with *Elav-Gal4* flies. Lifespan (**C**) and locomotor activity (**D**) of flies with indicated genotypes are shown.

**S4 Fig. *grh* is upregulated upon neuronal *Edis* depletion.** (**A**) RNA levels of genes encoding a series of temporal transcription factors in fly brain samples were measured by real-time PCR and normalized to control samples. *grh* was among the upregulated genes in *Edis*-depleted fly brain (n=6). (**B**) Overexpression of *grh* in the MB neurons led to MB morphology defects. *UAS-grh* or *UAS-mCD8GFP* (control) flies were crossed to *ok107-Gal4* flies. Brains of progeny of indicated genotypes were stained by anti-FasII antibody. MB morphology phenotypes of indicated genotypes were quantified. Chi-squared test was employed in statistical analysis. Sample numbers of each genotype are shown. (**C**) Various combinations of *UAS-shgfp*, *UAS-shmcherry*, *UAS-shEdis* and *UAS-shgrh* transgenes were crossed with *Elav-Gal4* flies. Levels of *Edis* and *grh* transcripts in flies of the indicated genotypes were measured by quantitative PCR (n=3). (**D**) Levels of *Edis*, *Relish*, and *grh* transcripts were measured in control *Elav>shgfp* or *Elav>shEdis* animals in wildtype, *Relish* heterozygous (*Rel^E20^*/+ or *Rel^E38^/+*) or homozygous (*Rel^E20/E38^*) mutant background (n=3). Levels of *grh* mRNA in *Elav>shEdis* flies are significantly lower in *Relish* homozygous (*Rel^E20/E38^*) compared with *Relish* heterozygous or wildtype background.

**S5 Fig. Relish binds to the promoter region of *castor* and induces MB morphology defects.** (**A**) Integrative genomics viewer (IGV) plot of Rel-N-GFP ChIP sequence result showing the Relish peaks at the promoter region of *castor*. (**B-C**) RelN overexpression in MB neurons led to MB morphology defects. *UAS-Flag-RelN* or *UAS-mCD8GFP* (control) flies were crossed to *Elav-Gal4* flies. Brains of progeny of indicated genotypes were stained by anti-FasII antibody (**B**). The scale bar indicates 50 µm. MB morphology phenotypes of indicated genotypes were quantified in **C**. Chi-squared test was employed in statistical analysis. Sample numbers and percentages of samples showing normal MB morphology in each genotype are shown.

**S1 Table. Differentially expressed genes upon *Edis* knockdown in neurons.** RNA-seq analysis was performed using RNA samples extracted from *Elav>shEdis* or *Elav>shGFP* (control) fly heads. Differential expressed genes (DEGs) in knockdown vs Control were defined using cut-off criteria of absolute fold change of >=2.0 and p value of =< 0.05.

**S1 Data. Numerical data underlying the figures.**

**S2 Data. Images underlying the figures.**

**Striking Image.** Single mushroom body neurons were labeled using the MACRM technique in control animals. Fas II (red) labels the mushroom body αβ axon bundles, and GFP (green) labels a single mushroom body αβ neuron that branched its axon dorsally along the α lobe and medially along the β lobe.

**The genotypes of the fly strains generated in the paper are as following:**

**Fig 1A-1D’**: *+/Y*. **1F, 1H and 1I**: *Elav-Gal4/ Y; UAS-shGFP/+*. *Elav-Gal4/Y; UAS-shEdis/+*. *Elav-Gal4/ Y; UAS-shGFP/+*. *Elav-Gal4/Y; UAS-shEdis/+*. *Elav-Gal4/Y; UAS-Dcr2/+; UAS-dsEct4/+*. *Elav-Gal4/Y; UAS-shEct4/+*. **1J and 1J’**: *yw, hsFlp, UAS-mCD8GFP/Y; FRT40A Tub-Gal80/ FRT40A UAS-mCD8GFP; UAS-shmcherry/+; ok107-Gal4/+.* **1K and 1K’**: *yw, hsFlp, UAS-mCD8GFP/Y; FRT40A Tub-Gal80/ FRT40A UAS-mCD8GFP; UAS-shEdis/+; ok107-Gal4/+.*

**Fig 2B**: *wor-Gal4/+; UAS-shGFP/+.* **2B’**: *wor-Gal4/+; UAS-shEdis/+.* **2C**: *UAS-shGFP/+; ok107-Gal4/+.* **2C’**: *UAS-shEdis/+; ok107-Gal4/+.***2D**: *GMR71C09-Gal4/UAS-shGFP.* **2D’**: *GMR71C09-Gal4/UAS-shEdis.* **2E**: *201Y-Gal4/+; UAS-shGFP/+.* **2E’**: *201Y-Gal4/+; UAS-shEdis/+.* **2F**: *c309-Gal4/+; UAS-shGFP/+.* **2F’**: *c309-Gal4/+; UAS-shEdis/+.* **2H, 2I, 2J and 2K**: *Elav GS-Gal4/ UAS-shGFP.* **2H’, 2I’, 2J’ and 2K’**: *Elav GS-Gal4/ UAS-shEdis.* **2M**: *Elav-Gal4/+; mb247-Gal80/+; UAS-shGFP/+.* **2M’**: *Elav-Gal4/+; mb247-Gal80/+; UAS-shEdis/+.* **2N**: *repo-Gal4/+; UAS-shGFP/+.* **2N’**: *repo-Gal4/+; UAS-shEdis/+.*

**Fig 3A and 3B**: *Elav-Gal4/+; UAS-Dcr2/+; UAS-shGFP/+. Elav-Gal4/+; UAS-Dcr2/+; UAS-shEdis/+. Elav-Gal4/+; UAS-Dcr2/+; UAS-shGFP/Rel^E20^. Elav-Gal4/+; UAS-Dcr2/+; UAS-shEdis/Rel^E20^. Elav-Gal4/+; UAS-Dcr2/+; UAS-shGFP/Rel^E38^. Elav-Gal4/+; UAS-Dcr2/+; UAS-shEdis/Rel^E38^. Elav-Gal4/+; UAS-Dcr2/+; UAS-shGFP Rel^E20^/Rel^E38^. Elav-Gal4/+; UAS-Dcr2/+; UAS-shEdis Rel^E20^/Rel^E38^.* **Fig. 3C and 3D:** *Elav-Gal4/+; UAS-Dcr2/+; UAS-shmcherry/UAS-shGFP.* *Elav-Gal4/+; UAS-Dcr2/+; UAS-shmcherry/UAS-shEdis. Elav-Gal4/+; UAS-Dcr2/+; UAS-shRel/UAS-shGFP. Elav-Gal4/+; UAS-Dcr2/+; UAS-shRel/UAS-shEdis.* **Fig. 3E**: *Elav-Gal4/+; UAS-Dcr2/+; UAS-shGFP/+. Elav-Gal4/+; UAS-Dcr2/+; UAS-shEdis/+. Elav-Gal4/+; UAS-Dcr2/+; UAS-shEdis/Rel^E20^. Elav-Gal4/+; UAS-Dcr2/+; UAS-shEdis/Rel^E38^. Elav-Gal4/+; UAS-Dcr2/+; UAS-shEdis Rel^E20^/Rel^E38^.*

**Fig 4A**: *Elav-Gal4/+; UAS-DptA/+. Elav-Gal4/+; UAS-Drs/+. Elav-Gal4/+; UAS-Dro/+. Elav-Gal4/+; UAS-Def/+.*

**Fig 5A and 5A’**: *Elav-Gal4/+; UAS-mCD8GFP/+.* **Fig 5B and 5C’**: *Elav-Gal4/+; UAS-castor-3xHA/+.* **Fig 5J and 5G**: *Elav-Gal4/+; UAS-Dcr2/+; UAS-shmcherry/UAS-shGFP.* *Elav-Gal4/+; UAS-Dcr2/+; UAS-shmcherry/UAS-shEdis. Elav-Gal4/+; UAS-Dcr2/+; UAS-shcastor/UAS-shEdis. Elav-Gal4/+; UAS-Dcr2/+; UAS-dscastor/UAS-shEdis.* **Fig 5K and 5G**: *Elav-Gal4/+; UAS-Dcr2/+; UAS-shmcherry/UAS-shGFP.* *Elav-Gal4/+; UAS-Dcr2/+; UAS-shmcherry/UAS-shEdis. Elav-Gal4/+; UAS-Dcr2/+; UAS-shgrh/UAS-shEdis.*

**Fig 6A**: *Elav-Gal4/+; UAS-Dcr2/+; UAS-shGFP/+.* *Elav-Gal4/+; UAS-Dcr2/+; UAS-shEdis/+.* *Elav-Gal4/+; UAS-Dcr2/+; UAS-shEdis/ Rel^E20^. Elav-Gal4/+; UAS-Dcr2/+; UAS-shEdis/ Rel^E38^. Elav-Gal4/Rel^E20^; UAS-Dcr2/+; UAS-shEdis Rel^E20^/ Rel^E38^.*

**S1A-S1F’ Fig**: *Elav-Gal4/+; UAS-shGFP/+. Elav-Gal4/+; UAS-shEdis/+.* **S1G Fig**: *Elav-Gal4/+; UAS-laccase2 vector/+; Elav-Gal4/+; UAS-laccase2 Edis.*

**S2 Fig**: *Elav-Gal4/+; UAS-shGFP/+. Elav-Gal4/+; UAS-shEdis/+.*

**S3A and S3B Fig**: *UAS-mCD8GFP/+; ok107-Gal4/+. UAS-castor-3XHA; ok107-Gal4/+.* **S3C and S3D Fig**: *Elav-Gal4/+; UAS-Dcr2/+; UAS-shmcherry/shGFP; Elav-Gal4/+; UAS-Dcr2/+; UAS-shEdis/shGFP; Elav-Gal4/+; UAS-Dcr2/+; UAS-dscastor/shEdis.*

**S4A Fig**: *Elav-Gal4/+; UAS-shGFP/+. Elav-Gal4/+; UAS-shEdis/+.* **S4B Fig**: ok107-Gal4/+. *UAS-grh/+*; *ok107Gal4/+*. **S4C Fig**: *Elav-Gal4/+; UAS-Dcr2/+; UAS-shmcherry/UAS-shGFP.* *Elav-Gal4/+; UAS-Dcr2/+; UAS-shmcherry/UAS-shEdis. Elav-Gal4/+; UAS-Dcr2/+; UAS-shgrh/UAS-shEdis.* **S4D Fig**: *Elav-Gal4/+; UAS-Dcr2/+; UAS-shGFP/+.* *Elav-Gal4/+; UAS-Dcr2/+; UAS-shEdis/+.* *Elav-Gal4/+; UAS-Dcr2/+; UAS-shEdis/ Rel^E20^. Elav-Gal4/+; UAS-Dcr2/+; UAS-shEdis/ Rel^E38^. Elav-Gal4/Rel^E20^; UAS-Dcr2/+; UAS-shEdis Rel^E20^/ Rel^E38^.*

**S5B and S5C Fig**: *Elav-Gal4/+; Elav-Gal4/UAS-RelN.*

*Lists of fly stocks*

| **Stock description** | **Stock number** | **Stock collection center** |
| --- | --- | --- |
| *UAS-shmcherry* |  | Zhou Lab |
| *UAS-shGFP* |  | Zhou Lab |
| *UAS-shEdis* |  | Zhou Lab |
| *UAS-flag-Edis-p* |  | Zhou Lab |
| *UAS-laccase2-Edis* |  | Zhou Lab |
| *UAS-DptA* |  | Zhou Lab |
| *UAS-Drs* |  | Zhou Lab |
| *UAS-Dro* |  | Zhou Lab |
| *UAS-Def* |  | Zhou Lab |
| *FRT 40A UAS-mCD8GFP; shEdis* |  | Zhou Lab |
| *FRT 40A UAS-mCD8GFP; shGFP* |  | Zhou Lab |
| *UAS-FlagRelN* |  | Gift of Dr. Neal Silverman |
| *w[1118]; P{w[+mC]=UAS-Dcr-2.D}2* | 24650 | Bloomington Drosophila stock center |
| *P{w[+mW.hs]=GawB}elav[C155]* | 458 | Bloomington Drosophila stock center |
| *P{ry[+t7.2]=hsFLP}1, y[1] w[*] P{w[+mC]=UAS-mCD8::GFP.L}Ptp4E[LL4]; P{w[+mC]=tubP-GAL80}LL10 P{ry[+t7.2]=neoFRT}40A; P{w[+mW.hs]=GawB}OK107 ey[OK107]* | 44406 | Bloomington Drosophila stock center |
| *y[1] v[1]; P{y[+t7.7] v[+t1.8]=TRiP.HMJ30091}attP40* | 63525 | Bloomington Drosophila stock center |
| [*y1*](https://flybase.org/reports/FBal0018607) [*v1*](https://flybase.org/reports/FBal0017656)*;*[*P{TRiP.JF01681}attP2*](https://flybase.org/reports/FBti0131046) | 31175 | Bloomington Drosophila stock center |
| *w[*]; P{w[+mC]=wor.GAL4.A}2; Dr[1]/TM3, P{w[+m*]=Ubx-lacZ.w[+]}TM3, Sb[1]* | 56553 | Bloomington Drosophila stock center |
| *w[1118]; P{y[+t7.7] w[+mC]=GMR71C09-GAL4}attP2* | 39575 | Bloomington Drosophila stock center |
| *w*; P{UAS-grh.B}v12M/CyO* | 42227 | Bloomington Drosophila stock center |
| y[1] sc[*] v[1] sev[21]; P{y[+t7.7] v[+t1.8]=TRiP.HMS02446}attP40 | 42611 | Bloomington Drosophila stock center |
| *y[1] w[67c23]; P{w[+mC]=UAS-mCD8::GFP.L}LL5 P{w[+mW.hs]=GawB}Tab2[201Y]* | *64296* | Bloomington Drosophila stock center |
| *w[*]; P{w[+mW.hs]=GawB}c309* | 6906 | Bloomington Drosophila stock center |
| *y[1] w[*]; P{w[+mC]=elav-Switch.O}GSG301* | 43642 | Bloomington Drosophila stock center |
| *y[1] w[67c23]; P{w[+m*]=mb247-GAL80.T}2* | *64306* | Bloomington Drosophila stock center |
| *w1118; P{GAL4} repo/TM3, Sb1* | *7415* | Bloomington Drosophila stock center |
| *w[*]; P{y[+t7.7] w[+mC]=10XUAS-IVS-mCD8::GFP}attP2* | *32185* | Bloomington Drosophila stock center |
| *y[1] v[1]; P{y[+t7.7] v[+t1.8]=TRiP.JF02083}attP2* | *26130* | Bloomington Drosophila stock center |
| *y[1] sc[*] v[1] sev[21]; P{y[+t7.7] v[+t1.8]=TRiP.HMS01180}attP2* | *34701* | Bloomington Drosophila stock center |
| *w[1118]; Rel[E20] e[s]* | 9457 | Bloomington Drosophila stock center |
| *w[1118]; Rel[E38] e[s]* | 9458 | Bloomington Drosophila stock center |
| [*y1*](http://flybase.org/reports/FBal0018607) [*v1*](http://flybase.org/reports/FBal0017656)*;*[*P{TRiP.HM05154}attP2*](http://flybase.org/reports/FBti0127770) | 28943 | Bloomington Drosophila stock center |
| *M{UAS-cas.ORF.3xHA.GW}ZH-86Fb* | 001826 | Zurich ORFeome Project |

List of primers

| **Primer names** | **Primer sequences** | **Assays** |
| --- | --- | --- |
| RpL32-F | ATCGGTTACGGATCGAACAA | RT-qPCR of mRNAs |
| RpL32-R | GACAATCTCCTTGCGCTTCT |  |
| Dpt-F | TTGCAGTCCAGGGTCACCAG |  |
| Dpt-R | TTCAGTCCAATCTCGTGGCG |  |
| Edis-F-873 | GGAGGAAACAACAAGACGAT |  |
| Edis-R-189 | GCATCATATTCTGCATGGAC |  |
| amos-PP1825F | CCAGCGATTCCCGAGTTCC |  |
| amos-PP1825R | CGGCGTATCGTAATACATCTCC |  |
| Lsp2-PP8577F | ATGAAGTCGTTCACGGTGATTG |  |
| Lsp2-PP8577R | GCTCCCACGGCTTGTACTC |  |
| abd-A-PP17996F | CACAGTTCGCTCAGTTCTATCAA |  |
| abd-A-PP17996R | TAAGCGTCATCCAGGGATACC |  |
| nos-PP28159F | CACCGCCAATTCGCTCCTTAT |  |
| nos-PP28159R | GCTGGTGACTCGCACTAGC |  |
| sna-PP7697F | ATGGCCGCCAACTACAAAAG |  |
| sna-PP7697R | GCAAACTGTGAGTCCTTGGTC |  |
| spo-PP11119F | TGGCGATTTTACTGAGTGTTCTG |  |
| spo-PP11119R | TCCTGGAGCCTGGGTATATTTTT |  |
| Poxm-PP30298F | ATGCGACAAAACGAATGTACCC |  |
| Poxm-PP30298R | GGGATTACCCAGATTGCTCAGA |  |
| ac-PP36651 | GCGCTTGCAGAAAGTTCTTCA |  |
| ac-PP36651R | AGGCGTATAAGTGTTGATGCTG |  |
| cas-PP4363F | GGCTGGATACACTTACACACAG |  |
| cas-PP4363R | CGCTTGTGCGTTCTTGTCTTG |  |
| gskt-PP6367F | ATGGCTTCCCAGAGTAAGAACA |  |
| gskt-PP6367R | ACAACTTTCGCGTCCGTGTA |  |
| wntD-PP13276F | TTTGCCATCACATTCTTCATGGG |  |
| wntD-PP13276R | GGGTGTACTGGTAGTAGCTCA |  |
| l(1)sc-PP19293F | TGTTGGACCCATGTTGTCCTC |  |
| l(1)sc-PP19293R | GTCTGGCTACCGATGGCAA |  |
| mud-PP36073F | TTCATTGCACAACAGGATGACA |  |
| mud-PP36073R | GCTTCTTAACGCACGAGTAATGC |  |
| brat-PP28711F | CAGCAATCTTCAGTCCTCAAACA |  |
| brat-PP28711R | GGAGATCCATTGGCGGATGT |  |
| Prospero-PP8567F | GCCCTGTTCCAACCACAATC |  |
| Prospero-PP8567R | CGAAACTGGTGAGTTGCTCG |  |
| numb-PP30545F | CGAGACCAAGGGCCTGATAG |  |
| numb-PP30545R | GGAAGCCGTGACACATCCAG |  |
| E(spl)m7-PP14789F | TTTTGGAACCACACTGATGACC |  |
| E(spl)m7-PP14789R | CCACAGACGATACTGAGTGGA |  |
| hb-PP7515F | CAGAACTGGGAGACGACAGC |  |
| hb-PP7515R | CCAGGTGATTGGTCGAGGG |  |
| svp-PP22994F | CACTCGTACCTCAGTTCCTACA |  |
| svp-PP22994R | TTGTCGATGCCCATAATGTTGT |  |
| Kr-PA60090F | AAACGCGAACCTTAGCTGCTG |  |
| Kr-PA60090R | GATAGCGACATGGAACGGTC |  |
| pdm2-PP11466F | GCGTTCGCTATCAGACTGC |  |
| pdm2-PP11466R | CCAGGACCTCGTTTTCCAGC |  |
| nub-PP36127F | GCCGGTTCCGGTAATTTCAGT |  |
| nub-PP36127R | AACGGCGAGATGCCACTAC |  |
| grh-PP1626F | GCTTCTACGGCCATGAGACTG |  |
| grh-PP1626R | GGCGGAAAATGGTTGTGGAAC |  |
| Atg1 (-723) F | GGCTCAATTAAGTAGTGTTACCAGA | For ChIP assay |
| Atg1 (-723) R | CCTAAAATGACGGTTTAAGCGGA |  |
| CHIP Diedel-F | TCTCTCCAGCGAACACAACA |  |
| CHIP Diedel-R | CCGCTCGCATTGTAATCGAA |  |
| CHIP DptA-801F | GATCCTGGTCATCATTGCCC |  |
| CHIP DptA-801R | ACTTTACAGCTGGGACACGT |  |
| CHIP castor-215F | GCGGGATTCCTTCACAAAATA |  |
| CHIP castor-215R | CAGGAAAATGCTCTGGGAAA |  |
| CHIP castor-1429F | CATTGCCGAGTAAAATGCAA |  |
| CHIP castor-1429R | AGCTCCTCCAGCCAGTGTTA |  |
| casluc-1924 WT- F | GGGGTACCCAACTGAGCTGGCCAGCAAC | for making overexpression constructs |
| casluc-1 WT- R | CCCAAGCTTAACTCTTCCGCGGCCTCC |  |
| casluc-215 KO F | AGAGCATTTTCCTGCGAAGG |  |
| casluc -215 KO R | AGCTTGTTTTCGGCTGGTTTC |  |
| casluc-1429 KO F | TTCCTGATTTGCATAAGTTTCCAG |  |
| casluc-1429 KO R | ATCGACGAATCGATTGCCGG |  |
| DptA F | GGAATTCATGCAGTTCACCATTGCC |  |
| DptA R | CCGCTCGAGTTAGAAATTCGGAAATCTGTAGG |  |
| Dro F | GGAATTCATGAAGTTCACCATCGTTTT |  |
| Dro R | CCGCTCGAGTTAGGCGGGCAGAATGG |  |
| Drs F | GGAATTCATGATGCAGATCAAGTACTT |  |
| Drs R | CCGCTCGAGTTAGCATCCTTCGCACCA |  |
| Def F | GGAATTCATGAAGTTCTTCGTTCTCG |  |
| Def F | CCGCTCGAGTCAATTGCGGCAAACGC |  |
